# Plant DNA methylation is sensitive to parent seed N content and influences the growth of rice

**DOI:** 10.1186/s12870-021-02953-3

**Published:** 2021-05-11

**Authors:** Xiaoru Fan, Laihua Liu, Kaiyun Qian, Jingguang Chen, Yuyue Zhang, Peng Xie, Man Xu, Zhi Hu, WenKai Yan, Yufeng Wu, Guohua Xu, Xiaorong Fan

**Affiliations:** 1 State Key Laboratory of Crop Genetics and Germplasm Enhancement, MOA Key Laboratory of Plant Nutrition and Fertilization in Low-Middle Reaches of the Yangtze River, Nanjing Agricultural University, Nanjing, 210095 China; 2 College of Resource and Environmental Science, Nanjing Agricultural University, Nanjing, 210095 China; 3 Vazyme Biotech Co Ltd, Nanjing, 210033 China; 4 School of Agriculture, Sun Yat-sen University, Guangzhou, 510275 China; 5 Bioinformatics Center, Nanjing Agricultural University, Nanjing, 210095 China

**Keywords:** Seed N content, Methylation, Phenotype, High N-use efficiency, OsNAR2.1

## Abstract

**Background:**

Nitrogen (N) is an important nutrient for plant growth, development, and agricultural production. Nitrogen stress could induce epigenetic changes in plants. In our research, overexpression of the *OsNAR2.1* line was used as a testing target in rice plants with high nitrogen-use efficiency to study the changes of rice methylation and growth in respond of the endogenous and external nitrogen stress.

**Results:**

Our results showed that external N deficiency could decrease seed N content and plant growth of the overexpression line. During the filial growth, we found that the low parent seed nitrogen (LPSN) in the overexpression line could lead to a decrease in the filial seed nitrogen content, total plant nitrogen content, yield, and *OsNAR2.1* expression (28, 35, 23, and 55%, respectively) compared with high parent seed nitrogen (HPSN) in high nitrogen external supply. However, such decreases were not observed in wild type. Furthermore, methylation sequencing results showed that LPSN caused massive gene methylation changes, which enriched in over 20 GO pathways in the filial overexpression line, and the expression of *OsNAR2.1* in LPSN filial overexpression plants was significantly reduced compared to HPSN filial plants in high external N, which was not shown in wild type.

**Conclusions:**

We suggest that the parent seed nitrogen content decreased induced DNA methylation changes at the epigenetic level and significantly decreased the expression of *OsNAR2.1*, resulting in a heritable phenotype of N deficiency over two generations of the overexpression line.

**Supplementary Information:**

The online version contains supplementary material available at 10.1186/s12870-021-02953-3.

## Background

Rice (*Oryza sativa* L.) is a major staple food for a large part of the global population. Nitrogen (N) is an essential macronutrient for plant growth and crop productivity [1]. Rice cultivation requires higher inputs of nitrogen fertilizers than other crops [2, 3]. Nitrogen deficiency is severe environmental stress that can impair crop production and prevent plants from effectively absorbing enough nitrogen for sufficient growth [4]. There have been previous reports about the reduction of seed N concentrations due to filial nitrogen deficiency in many species including rice [5], soybean [6], pea [7], and cotton [8]. Growth restrictions were greater with lower seed N when the seedlings were exposed to solutions without external N in soybean [9]. Nitrogen fertilizer on parent plants could improve the salinity tolerance of filial wheat during germination and the early seedling growth stages [10]. Also, seed nitrogen enhances seed vigor and accelerates germination time in rice [11]. However, there is little research on the influence of seed nitrogen on the growth of mature rice in the field.

Epigenetics is defined as the study of DNA sequence-independent changes in gene function that are mitotically and/or meiotically heritable [12]. DNA methylation is the major heritable epigenetic modification and contributes to the epigenetic regulation of nuclear gene expression and genome stability [13]. The cytosine residue in DNA is methylated at the 5′ position [14] and occurs throughout the plant genome, including CG, CHG, and CHH (H = A, T, or C) [15]. There are many studies of epigenetic regulation in plant response to nutrient stresses [16–20]. There have been reports that phosphate starvation could result in extensive remodeling of global DNA methylation in a plant and is often associated with changes in gene expression [21–24]. Zinc deficiency can lead to hypo- and hyper-methylated chromosomal regions [17]. Nitrogen-deficiency stress can induce heritable alteration in DNA methylation [19]. Fan et al. (2020) reported that overexpression of *OsNAR2.1* does change rice DNA methylation status [25]. However, the research progress regarding epigenetic influence by the nutrient in both external and endogenous seed is very sparse.

OsNAR2.1 is a high-affinity nitrate transporter partner protein which, along with OsNRT2s, plays a central role in nitrate absorption and translocation in rice [26–30]. Previous reports have demonstrated that using *OsNAR2.1* promoter instead of ubiquitin promoter to drive *OsNRT2.1* can improve the agronomic nitrogen use efficiency and yield in rice [30]. Chen et al. (2017) showed that using *OsNAR2.1* native promoter to drive *OsNAR2.1* (*pOsNAR2.1:OsNAR2.1*) improves nitrate and ammonium absorption and increases nitrogen-use efficiency (NUE) in rice cultivation systems [27]. We used *pOsNAR2.1:OsNAR2.1* (Ox1) in Chen et al. (2017) as a testing high N-use efficiency rice and to explore both seed and external N content influence on the growth of rice.

Our research aims to elucidate the influence of seed N resource on the wild type and high N-use efficiency Ox1 line growth in the both seedling and mature, and the methylation change in response to the different seed N and different external N resource.

## Results

### Nitrogen deficiency could cause a heritable decrease in the growth of *OsNAR2.1 *overexpression line

**Fig. 1:**
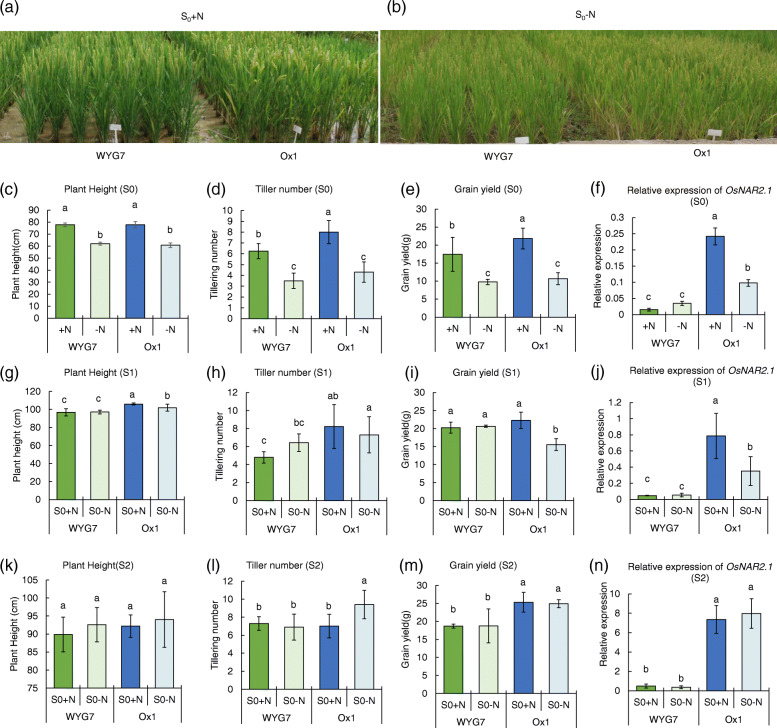
Nitrogen deficiency in S0 generation results in the decrease of plant height and yield of WYG7 and Ox1 and heritable in S1 generation in Ox1. (**a, b**) Gross morphology of WYG7 and Ox1 in +N and -N field in S0 generation. Bar = 20 cm. (**c**) Plant height and (**d**) Tiller number of WYG7 and Ox1 in +N and -N field in S0 generation. Error bar: SD (*n* = 10 plants). (**e**) Grain yield per plant of WYG7 and Ox1 in +N and -N field in S0 generation. Error bar: SD (*n* = 5 plants). (**f**) Relative expression of *OsNAR2.1* in WYG7 and Ox1 in +N and -N field in S0 generation. Error bar: SD (*n* = 3 plants). (**g**) Plant height and (**h**) Tiller number of WYG7 and Ox1 in S1 generation. Error bar: SD (n = 10 plants). (**i**) Grain yield per plant of WYG7 and Ox1 in S1 generation. Error bar: SD (n = 5 plants). (**j**) Relative expression of *OsNAR2.1* in WYG7 and Ox1 in S1 generation. Error bar: SD (n = 3 plants). (**k**) Plant height and (**l**) Tiller number of WYG7 and Ox1 in S2 generation. Error bar: SD (n = 10 plants). (**m**) Grain yield per plant of WYG7 and Ox1 in S2 generation Error bar: SD (n = 5 plants). (**n**) Relative expression of *OsNAR2.1* in WYG7 and Ox1 in S2 generation. Error bar: SD (n = 3 plants). Significant differences between different lines are indicated by different letters (*P* < 0.05, one-way ANOVA). WYG7: wild type of Wuyungeng 7; Ox1: overexpression of *OsNAR2.1* by *OsNAR2.1* promoter; S0 + N: nitrogen treatment with 300 kg N/ha in S0 generation; S0-N: nitrogen treatment with 0 kg N/ha in the S0 generation

We planted overexpression lines of *OsNAR2.1 *(Ox1) and wild type of Wuyungeng 7(WYG7) in the field with 300 kg N/ha (+N) and 0 kg N/ha (−N) (Fig. 1a, b) in the first-generation (S0 generation). In the S0 generation, the plant height of WYG7 and Ox1 decreased by 20 and 22%, the tiller number decreased by 44 and 46%, and the grain yield per plant decreased by 44 and 51%, respectively, in the -N field compared with +N (Fig. 1c-e). Meanwhile, the relative expression of *OsNAR2.1* in Ox1 decreased by 62% in the -N field, but a significant difference was not observed in WYG7 between the +N and -N field (Fig. 1f). Other agronomic traits showed that nitrogen deficiency in S0 generation reduced the panicle length of WYG7 and Ox1 by 21 and 14% and the seed setting rate decreased by 7 and 18%, respectively. However, there was no difference in the grain weight per panicle, the number of grains per panicle, and the 1000-grain weight in WYG7 or Ox1 between the +N and -N field (Table S1).

The seeds were harvested from the +N and -N fields in the S0 generation and planted in the field restored with +N for the S1 and S2 generations. We found that when the nitrogen treatment was restored to +N in the S1 generation, the nitrogen deficiency in the S0 generation did not influence the plant height, tiller number, and grain yield of WYG7 in the S1 generation (Fig. 1g-i). However, under the conditions of high nitrogen fertilizer in the S1 generation, nitrogen deficiency in the S0 generation caused the plant height to be reduced by 4%, the grain yield per plant reduced by 20%, while the tiller number displayed no significant change in Ox1 line (Fig. 1g-i). Also, nitrogen deficiency in the S0 generation caused downregulation of *OsNAR2.1* of 55% in the Ox1 line in S1 (Fig. 1j), a decrease in the panicle length 7%, a reduction in the grain weight per panicle by 27%, and a reduction of the seed setting rate by 10% (Table S2) in S1 generation.

However, under continued high nitrogen treatment in the S2 generation, we found that the S0 generation nitrogen deficiency did not affect the plant height, grain yield, and *OsNAR2.1* expression of the wild type and Ox1 line in the S2 generation. The tiller number of Ox1 treated with nitrogen deficiency in the S0 generation increased in the S2 generation (Fig. 1k-n). This was inconsistent with the changes in the tiller number in the S0 and S1 generations, therefore the change of tiller number in the S2 generation is related to the difference of the current environment and planting density.

In conclusion, nitrogen deficiency in the S0 generation caused a decrease in the growth of high N-use efficiency rice Ox1 line, and the decrease was inherited in the S1 generation despite the restoration of high nitrogen supply conditions. However, the decrease did not persist in the S2 generation.

### Parent N content decrease retards seedling growth in high nitrogen-use efficiency line

**Fig. 2:**
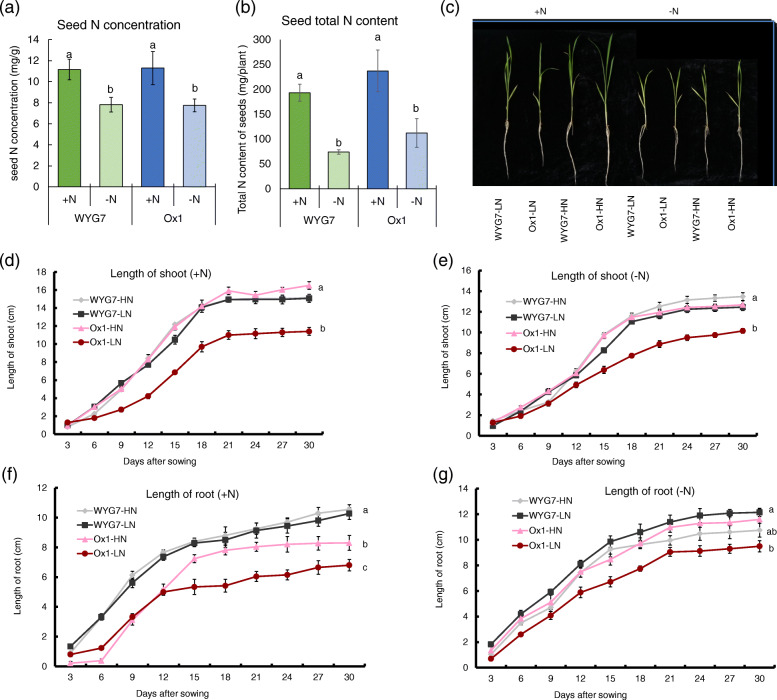
Parent seed nitrogen content retard filial seedling growth in Ox1 line. (**a**) Seed N concentration and (**b**) Seed total N content of WYG7 and Ox1 in +N and -N field. Error bar: SD (n = 5 plants). **c** Gross morphology of different lines in +N and -N water. Bar = 5 cm. **d, e** Track growth of shoot in 30 days of different lines in +N and -N water. Error bars: SD (n = 5 plants). **f, g** Track growth of root in 30 days of different lines in +N and -N water. Error bars: SD (n = 5 plants). Significant differences between different lines are indicated by different letters (*P* < 0.05, one-way ANOVA,). +N: water with 2.5 mM NH_4_NO_3_; −N: water with no N. WYG7-HN: WYG7 with high parent seed N content; WYG7-LN: WYG7 with low parent seed N content; Ox1-HN: Ox1 line with high parent seed N content; Ox1-LN: Ox1 line with low parent seed N content

Previous reports have shown that the reduced nitrogen content in seeds is endogenous nitrogen stress for plants, which also affects filial plant growth [9]. We found that field N deficiency in the S0 generation caused seed N concentration to be reduced by 30 and 32% and seed total N content to be reduced by 62 and 53%, in WYG7 and Ox1 line, respectively (Fig. 2a,b).

**Fig. 3:**
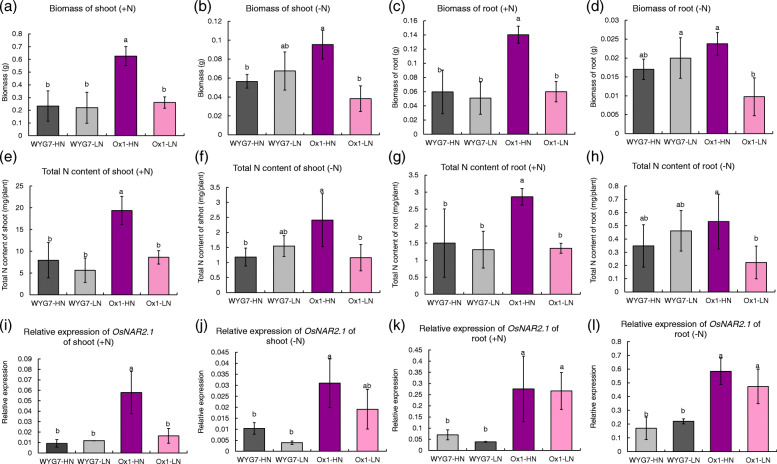
Parent seed nitrogen content decrease leads to the decrease of the filial seedling biomass, seedling nitrogen content and *OsNAR2.1 *expression in Ox1. **a, b** Biomass of shoot and at 30 day of different lines in +N and -N water. Error bars: SD (n = 5 plants). **c, d** Biomass of root at 30 day of different lines in +N and -N water. Error bars: SD (n = 5 plants). **e, f** Total N content of shoot at 30 day of different lines in +N and -N water. Error bars: SD (n = 5 plants). **g, h** Total N content of root at 30 day of different lines in +N and -N water. Error bars: SD (n = 5 plants). **i, j** Relative expression of* OsNAR2.1* of shoot in +N and -N water. Error bars: SD (*n* = 4 plants). **k, l** Relative expression of *OsNAR2.1* of root in +N and -N water. Error bars: SD (n = 4 plants). Significant differences between different lines are indicated by different letters (P < 0.05, one-way ANOVA)

In order to analyze the influence of the seed nitrogen on filial plant growth, we collected WYG7 and Ox1 seeds in the S0 generation from +N and -N fields (Fig. 1a,b). The Ox1 seeds collected from the +N field with high N content were labelled as Ox1-HN and seeds collected from -N field with low N content as Ox1-LN. The same labeling was done in WYG7, seeds of WYG7 collected from +N and -N fields were named WYG7-HN and WYG7-LN, respectively. For distinction, the seeds harvested from the S0 generation were named parent seed and the generated seeds in S1 generation were named as the filial seed. We grew four plants in water with NH_4_NO_3_ (+N) and water (−N) for 1 month (Fig. 2c) and found that there were no differences in the length of seedling shoots or roots between WYG7-HN and WYG7-LN during the 30 days (Fig. 2d-g). However, the length of the shoots in the Ox1-LN seedlings was significantly shorter compared with Ox1-HN in both +N and -N treatments since day 15 (Fig. 2d, e). The same pattern was observed in the roots (Fig. 2f, g). In the +N condition, the biomass and total N content of Ox-LN significantly decreased by 58 and 55% in the shoot (Fig. 3a, b) and decreased significant by 57 and 58% in the root, respectively, compared with Ox1-HN (Fig. 3c, d). In the -N condition, the biomass and total N content of Ox-LN significantly decreased in the shoot by 59 and 53% (Fig. 3e, f) and by 55 and 58% in the root, respectively, compared with Ox1-HN (Fig. 3g, h). We also tested the expression of *OsNAR2.1* and found that expression in Ox1-HN shoot was 3.5 times higher than in Ox1-LN and significantly higher in the +N treatment (Fig. 3i). In the -N treatment, the expression of *OsNAR2.1* in Ox1-HN shoot was 1.5 times higher compared with Ox1-LN, but not significant (Fig. 3j). At the same time, in both +N and –N conditions, there was no difference in the expression of *OsNAR2.1* in the root between Ox1-HN and Ox1-LN (Fig. 3k, l). However, in both +N and -N conditions, the biomass and total N content of WYG7-LN displayed no difference compared to WYG7-HN in either the shoot or the root (Fig. 3a-h). The expression of *OsNAR2.1* in WYG7-LN and WYG7-HN was significantly lower compared with Ox1(−LN and -HN) in both treatments but showed no difference between each other (Fig. 3i-l). Since OsNAR2.1 is a partner protein of OsNRT2.1 and OsNRT2.3a in the absorption of nitrate [26, 29], we detected the expression of both *OsNRT2.1* and *OsNRT2.3a* in Fig. S1. The results showed that in both +N and –N condition, the expression of *OsNRT2.1* and *OsNRT2.3a* in both shoot and root all have no difference between WYG-HN and WYG-LN (Fig. S1 a-h). But the expression of *OsNRT2.1* and *OsNRT2.3a* in Ox1-HN were significant higher than in Ox1-LN, WYG7-HN and WYG7-LN in shoot under +N condition (Fig. S1 a, c), which is similar with the expression of *OsNAR2.1* in +N condition in shoot.

In conclusion, the low parent seed nitrogen (LPSN) could lead to the retard of the growth and decrease of biomass, N content of seedling and decrease of *OsNAR2.1* expression of filial high N-use efficiency rice Ox1 line in seedlings, compared with high parent seed nitrogen (HPSN).

### Parent seed N content decrease leads to grain yield and N content decrease in *OsNAR2.1* overexpression line in HN fertilizer field

**Fig. 4:**
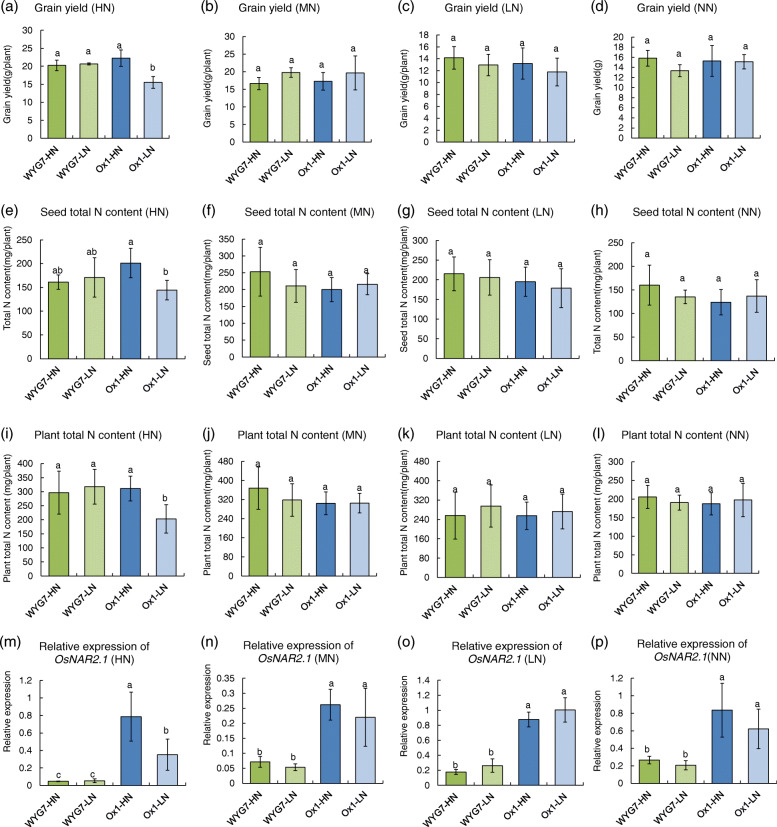
The decrease in the parent seed nitrogen content leads to the decrease of filial grain yield and N content of Ox1 in HN field. **a-d** Grain yield per plant of different lines in HN, MN, LN and NN fertilizer fields. Error bars: SD (n = 5). **e-h** Filial seed total N content of different lines in HN, MN, LN and NN fertilizer field. Error bars: SD (n = 5). **i-l** Plant total N content of different lines in HN, MN, LN and NN fertilizer fields. Error bars: SD (n = 5). **m-p** Relative expression of *OsNAR2.1* of different lines in HN, MN, LN and NN fertilizer fields. Error bars: SD (n = 4). Significant differences between different lines are indicated by different letters (P < 0.05, one-way ANOVA). HN: N fertilizer with 300 kg N/ha; MN: N fertilizer with 150 kg N/ha; LN: N fertilizer with 75 kg N/ha; NN: N fertilizer with 0 kg N/ha

We grew WYG7-HN, WYG7-LN, Ox1-HN, Ox1-LN lines in the field with four different N content: 300 (HN), 150 (MN), 75 (LN), and 0 kg N/ha (NN) (Fig. S2a-d). First, without endogenous seed N stress, the field N deficiency could induce 21 and 31% decrease of grain yield, and 23 and 32% of plant height in WYG-HN and Ox1-HN, respectively, in S1 generation, which is similar with as S0 generation (Fig. S3). Furthermore, we found that in Ox1-LN the plant height and the grain yield significantly decreased by 7 and 23%, respectively, compared to Ox1-HN in the HN field (Fig. S2e, Fig. 4a). No difference was observed in the MN, LN, and NN fields (Fig. S2f-h, Fig. 4b-d). Both the plant height and grain yield did not display a difference between WYG7-HN and WYG7-LN in all HN, MN, LN, and NN fields (Fig. S2e-h, Fig. 4a-d). The tiller number and chlorophyll content were not different between Ox1-LN and Ox1-HN and also not different between WYG7-LN and WYG7-HN in the four N treatment fields (Fig. S2i-l, S4a-d), and were not influenced by LPSN. Also, other agronomic traits showed that, in HN conditions, the panicle length, grain weight per panicle, and seed setting rate of Ox1-LN significantly decreased by 7, 27, and 10%, respectively, compared with Ox1-HN. However, in the MN, LN, and NN fields, these agronomic traits were not affected by the LPSN in Ox1. At the same time, in four fields, the panicle length, grain weight per panicle, seed setting rate, the number of seeds per panicle, and 1000-grain weight of WYG7 were not affected by the LPSN (Table S3).

Furthermore, we analyzed the filial seed N concentration, seed total N content, and plant total N content of Ox1-LN and found a significant reduction of 26, 28, and 35%, respectively, compared with Ox1-HN in the HN field (Fig. S4e and 4e, i). There were no significant differences in the MN, LN, and NN fields (Fig. S4e-h and 4e-l). For WYG7, there was no significant difference in the filial seed N concentration, seed total N content, and plant total N content between WYG7-HN and WYG7-LN in all HN, MN, LN, and NN fields (Fig. S4e-h, Fig. 4e-l). Simultaneously, we analyzed the expression of *OsNAR2.1* of four plants in the mature stage and found out that the expression of *OsNAR2.1* was significantly lower in the WYG7 lines compared to Ox1 lines. However, there was no difference between WYG7-HN and WYG7-LN in all four fields (Fig. 4m-p). In the HN field, the expression of *OsNAR2.1* was approximately 4.3 times higher in Ox1-HN than in Ox1-LN (Fig. 4m). But in the MN, LN, and NN fields, the expression of *OsNAR2.1* was not different between Ox1-HN and Ox1-LN (Fig. 4n-p). To confirm the phenotype causing LPSN, the various N content field experiment was repeated for one more year (the data presented in Table S4). We found the same pattern in plant growth in the third year compared with the second year, where the plant height, grain yield, filial seed N concentration, seed N content, plant N content, and expression of *OsNAR2.1* all were significantly reduced in Ox1-LN compared with Ox1-HN in HN condition but not in MN, LN, and NN treatments.

**Fig. 5:**
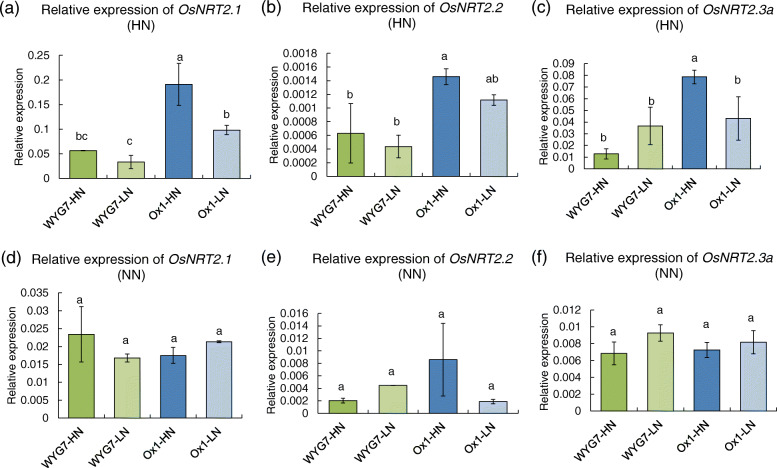
The decrease in the parent seed nitrogen content leads to the decrease of relative expression of *OsNRT2.1* and *OsNRT2.3a* of *OsNAR2.1* overexpression line in HN field. **a-c** Relative expression of *OsNRT2.1*, *OsNRT2.2* and *OsNRT2.3a* of different lines in high N fertilizer field. **d-f** Relative expression of *OsNRT2.1*, *OsNRT2.2* and *OsNRT2.3a* of different lines in no N fertilizer field. Significant differences between different lines are indicated by different letters (P < 0.05, one-way ANOVA, Duncan)

The OsNRT2 family proteins OsNRT2.1, OsNRT2.2, and OsNRT2.3a require the participation of OsNAR2.1 in the absorption of nitrate [26, 29] and the expression of *OsNAR2.1* are influenced by LPSN in Ox1 line. We, therefore, tested the expression of the *OsNRT2.1*, *OsNRT2.2* and *OsNRT2.3a*. In the HN condition, the expression of *OsNRT2.1* and *OsNRT2.3* significantly decreased by about 48 and 45%, respectively, in Ox1-LN compared with Ox1-HN (Fig. 5a, c). However, the expression of *OsNRT2.1*, *OsNRT2.2,* and *OsNRT2.3a* in Ox1-LN was not significantly different from that of WYG7-HN and WYG7-LN (Fig. 5a-c). Under NN conditions, the expression of *OsNRT2.1*, *OsNRT2.2,* and *OsNRT2.3a* was not significantly different between Ox1-HN, Ox1-LN, WYG7-HN, and WYG-LN lines (Fig. 5d-f). Therefore, compared with Ox-HN, LPSN of Ox-LN not only reduced the expression of *OsNAR2.1* but also reduced the expression of the *OsNRT2.1* and *OsNRT2.3a*.

In conclusion, when the field is sufficiently fertilized with nitrogen, LPSN could lead to the decrease of plant growth, grain yield, and expression of *OsNAR2.1*.

### Parent seed N content decrease leads to global methylation changes

**Fig. 6:**
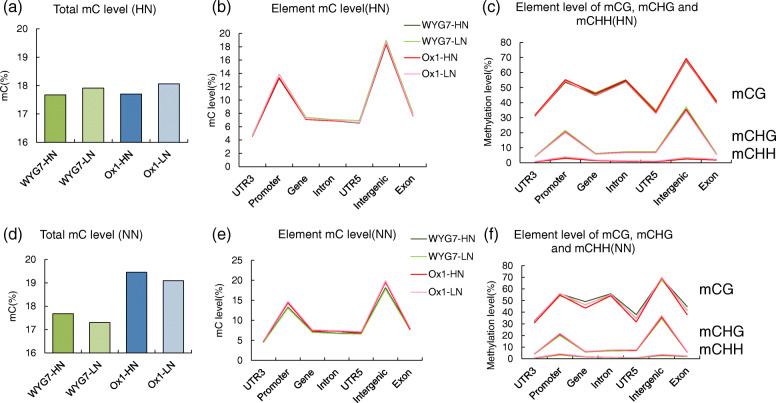
Methylation level for Ox1 and WYG7 lines with different parent N content seeds in different N fertilizer fields. **a** Total mC levelof different lines in HN field. **b** Element mC level of WYG7-HN, WYG7-LN, Ox1-HN and Ox1-LN in HN field. **c** Element level of mCG, mCHG, mCHH of different lines in HN field. **d** Total mC level of different lines in NN field. **e** Element mC level of of different lines in NN field. **f** Element level of mCG, mCHG, mCHH of of different lines in NN field

Since the genome of Ox1 and WYG7 did not change during a single generation but the phenotype of Ox1 was influenced by LPSN, we suspected that the epigenetic changes occurred between generations with different N treatments. We sequence the methylation status of WYG7-HN, WYG7-LN, Ox1-HN, Ox1-LN lines in the HN and NN treatment fields. The description, number of clean sequencing reads, valid C numbers, methylation C counts, and the percentages of mCs in CG, CHG, and CHH are presented in Table S5. The mCG ratio ranged from 52 to 56% and the non-mCG ratio was from 44 to 48%, similar to Li et al. (2015). Compared with HPSN, in the HN field, LPSN filial WYG7 and Ox1 lines showed lower methylation levels (Fig. 6a). However, LPSN filial WYG7 and Ox1 had higher methylation levels in the NN field (Fig. 6d). Furthermore, in the HN field, the methylation levels between WYG7-HN and Ox1-HN, WYG-LN and Ox1-LN were similar, with a difference of approximately 0.1 and 0.8%, respectively (Fig. 6a). In the NN field, the methylation level of Ox1-HN and Ox1-LN increased by around 10%, compared with WYG7-HN and WTG7-LN, respectively (Fig. 6d). The methylation level of three contexts in WYG7-LN all increased in HN and decrease in NN compared with WYG7-HN, similar to total mC level change (Fig. S5a-c, e-g).

Then we analyze the methylation level of different gene elements and found that, for the HN condition, the methylation level of different C context all showed no differences between four samples (Fig. 6b, c). However, the methylation level of global mC was higher in Ox1-HN and Ox1-LN in the promoter and intergenic regions compared with WYG7-HN and WYG7-LN in the NN condition. Moreover, the methylation level of CG of WYG7-HN was higher than WYG7-LN and Ox1-LN, and higher than in Ox1-HN in gene and 5’UTR regions (Fig. 6e, f). These results suggest that the global methylation level of different elements did not significantly change between Ox1 and WYG7 in the N fertilizer condition. However, the global methylation increased in Ox1 compared with WYG7, in N deficiency condition, especially in the promoter and intergenic regions. The methylation of mCG in gene and 5’UTR regions decreased in Ox1 compared with WYG7. Furthermore, we analyzed methylation level of enhancers in WYG7 and Ox1 lines, and found that in HN condition, the methylation levels of enhancer are increase 1.6 and 2.9% in WYG-LN and Ox1-HN, compared with WYG-LN and Ox1-LN, respectively. In NN condition, the methylation levels of enhancer are increase 1.5 and 6.7% in WYG-LN and Ox1-HN, compared with WYG-LN and Ox1-LN, respectively (Fig. S5d, h). The results indicated that the LPSN influence on enhancer is stronger in Ox1 under NN condition.

### Parent seed N content decrease induces more hypo-DMRs in the N fertilizer field but more hyper-DMRs in the N deficiency field in the overexpression line

**Fig. 7:**
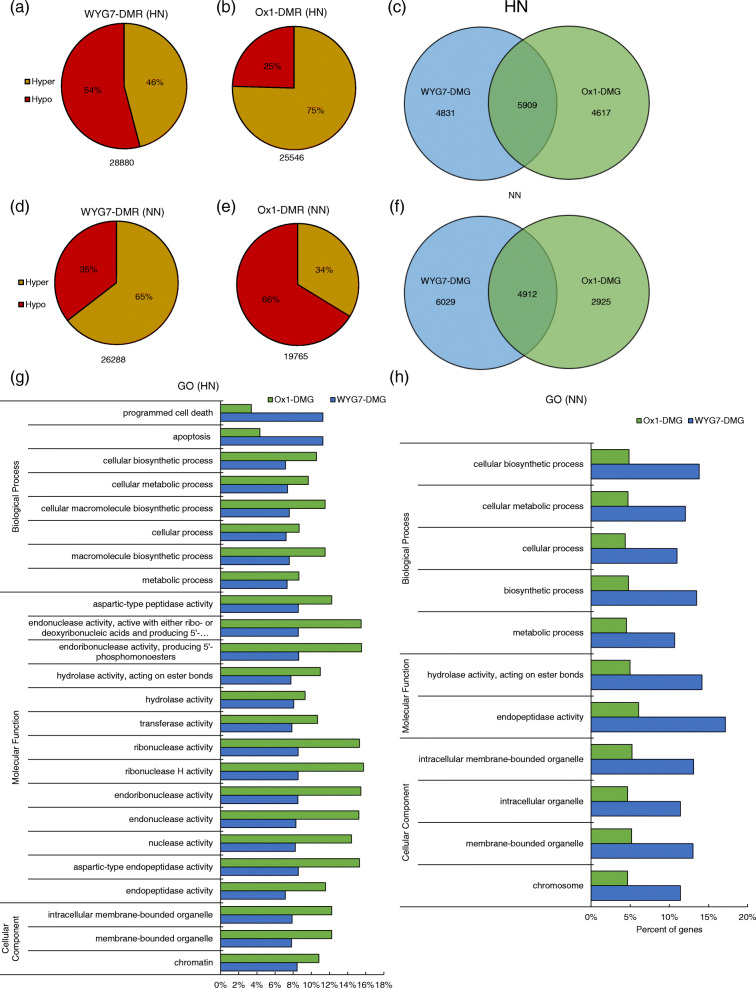
Parent seed N content decrease induced differential methylation in different N fertilizer fields. **a** Breakdown of hyper and hypo of WYG7-DMR in HN field. **b** Breakdown of hyper and hypo of Ox1-DMR in HN field. **c** Venn diagram of unique and shared DMGs between WYG7-DMG and Ox1-DMG in HN field. **d** Breakdown of hyper and hypo of WYG7-DMR in NN field. **e** Breakdown of hyper and hypo of Ox1-DMR in NN field. **f** Venn diagram of unique and shared DMGs in WYG7 and Ox1 in NN field. **g** GO cluster analysis of unique DMGs betweenWYG7-DMG and Ox1-DMG in HN field. **h** GO cluster analysis of unique DMGs in WYG7 and Ox1 in NN field. DMR: Differentially methylated region. DMG: Differentially methylated gene. WYG7-DMR: DMR between WYG7-LN and WYG7-HN; Ox1-DMR: DMR between Ox1-LN and Ox1-HN. ↑ indicate hyper-DMR; ↓ indicate hypo-DMR. WYG7-DMG: DMG between WYG7-LN and WYG7-HN; Ox1-DMG: DMG between Ox1-LN and Ox1-HN

We analyzed differentially-methylated regions (DMRs) between WYG7-LN and WYG7-HN, Ox1-LN and Ox1-HN, as WYG7-DMR and Ox1-DMR, respectively, in HN and NN treatments. We found that in HN fields, the WYG7-DMR was 28,880, and approximately 3400 more than 25,546 Ox1-DMR. However, WYG7-DMR was 26,288, 6500 more than 19,765 Ox1-DMR in the NN condition. Meanwhile, there are 46% hyper WYG7-DMRs in the HN field but, 65% hyper WYG7-DMRs in the NN field. On the other hand, 75% Ox1-DMRs are hyper in the HN condition but only 34% are hyper in the NN condition (Fig. 7a, b, d, e). We concluded that compare with HPSN, LPSN decreases causing more hypo-DMRs in the HN field but more hyper-DMRs in the NN field for WYG7, and in contract, causing more hyper-DMRs in the HN field but more hypo-DMRs in the NN field for Ox1 line.

Based on the annotation results of the DMRs on the genome, genes with DMRs in gene or promoter regions were defined as differentially methylated genes (DMGs). Both WYG7 and Ox1 lines had over 10,000 DMGs in the HN condition. In the NN treatment, there were over 10,000 DMGs in WYG7, but only around 8000 DMGs in Ox1 (Fig. 7c, f). The Venn results of DMGs showed that, in the HN condition, there were 5909 mutual DMGs between WYG7 and Ox1 more than 4831 unique DMGs in WYG7 and 4617 unique DMGs in Ox1. However, in the NN condition, there were 4912 mutual DMGs between WYG7 and Ox1 more than 2925 unique DMGs in Ox1, but less than 6029 unique DMGs in WYG7. In conclusion, LPSN caused over 10,000 DMGs in both WYG7 and Ox1 lines, and the majority of the DMGs were mutual in the HN field. However, in NN condition, LPSN caused over 10,000 DMGs in WYG7 and only around 8000 DMGs in Ox1. The majority of the DMGs in WYG7 were specific, but only a minority of the DMGs in Ox1 were specific. We hypothesized that in the NN condition, LPSN influence on gene methylation status in WYG7 was stronger than in Ox1.

Furthermore, we analyzed field N deficiency induced DMRs and DMGs of WYG7-HN and Ox1-HN lines (Fig. S6), without the interference of endogenous N deficiency. We found that the field N deficiency influence on WYG7 and Ox1 were also opposite. More than half (83%) of DMR in WYG7-HN causing by field N deficiency is hypo, and only 17% hyper-DMRs. In Ox1-HN lines, 91% hyper-DMRs and 9% hypo-DMRs induce by field N deficiency (Fig. 6a, b). The results were similar with LPSN influence on WYG7 and Ox1 in HN condition. Field N deficiency and seed N deficiency both induce more hypo-DMRs in WYG7 and more hyper-DMRs in Ox1 line. The Venn results of DMGs showed that, there were 7800 mutual DMGs between WYG7-HN and Ox1-HN induce by field N deficiency and 3813 unique DMGs in WYG7 and 5214 unique DMGs in Ox1 (Fig. S6c). The majority of the DMGs were mutual in WYG7-HN and Ox1-HN, also similar with LPSN induced DMGs between WYG7-DMG and Ox1-DMG in HN condition (Fig. 7c).

### Global DNA methylation patterns of genes were altered by parent seed N content and sensitive to field N content

In the HN condition, there were 4831 unique DMGs in the WYG7 line and 4617 unique DMGs in the Ox1 line. In the NN condition, there were 6029 unique DMGs in WYG7 and 2925 unique DMGs in Ox1 (Fig. 7c, f). We cluster analyzed the gene ontology enrichment (GO) of the unique DMGs (Fig. 7g, h). Based on their specific processes, these genes were divided into three major groups including biological process, cellular components, and molecular function. Twenty-two pathways were significantly enriched by unique Ox1-DMG, rather than WYG7-DMG, including six pathways in the biological process, thirteen pathways in molecular functions and three pathways in the cell component, containing cellular biosynthetic process, aspartic-type peptidase activity and intracellular, etc. Only two pathways were detected including programmed cell death and apoptosis, in which unique WYG7-DMGs were significantly enriched and Ox1-DMGs were not (Fig. 7g). Under NN conditions, there were 11 pathways where WYG7-DMG was significantly enriched, but Ox1 was not, including five pathways in the biological processes, two pathways in the molecular functions and four pathways in the cell component containing cell biosynthetic process, hydrolase activity and membrane-bounded organelle, etc. (Fig. 7h). But the pathway that unique Ox1-DMG significantly enriched were all enriched by unique WYG7-DMG. (Fig. 7g, h).

We choose some genes from DMG results and used Integrative Genomics Viewer (IGV) visualized their methylation states (Fig. S7 and S8). The genes we chosen include MADS family genes that modulates nitrate translocation in rice [31]; WAKY family genes that influence rice phosphorus accumulation [32]; amino acid transporters ATL family genes [33]; PHYB family genes that responds to starvation [34]; ammonium transporter AMT family genes [35], and some genes relative to methylation and miRNA regulations [36, 37]. Many research showed that gene methylation is highly correlated with transcription levels [38]. Therefore, we analyzed expression of these genes by quantitative reverse transcription PCR (qRT-PCR) (Fig. S7, 8). The results showed that in 12 genes, the expression of 7 genes were influence by methylation changes on gene body and promoter, including *OsMADS95, OsATL15, OsWAKY46* (Fig. S7b)*, OsMADS59, OsATL16, OsATM4* and *OsDRM1b* (Fig. S8b), and the expression of 7 genes all up-regulated by hypo-methylation.

We also visualized the methylation of the *OsNAR2.1* and *OsNRT* family genes in Fig. S9, which displayed no methylation difference between four lines in HN and NN conditions.

### DNA methyltransferases expression influenced by parent seed N content

**Fig. 8:**
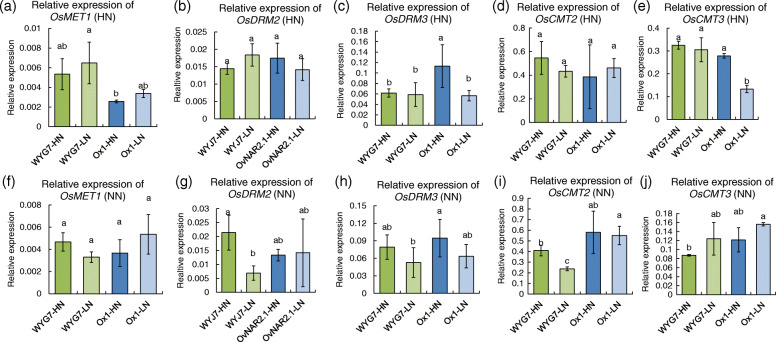
Relative expression of methyltransferases for WYG7 and Ox1 lines with different parent seed N content in different N fertilizer fields. **a-e** Relative expression of *OsMET1, OsDRM2, OsDRM3, OsCMT2* and *OsCMT3* in different lines in HN field. **f-j** Relative expression of *OsMET1, OsDRM2, OsDRM3, OsCMT2* and *OsCMT3* in different lines in NN field. Error bars: SD (n = 4 plants). Significant differences between different lines are indicated by different letters (P < 0.05, one-way ANOVA)

The change in total mC levels and the massive differential methylation regions in response to LPSN and field N content suggested that some of the genes encoding components of the DNA methylation machinery could be differentially regulated. To test this hypothesis, we used qRT-PCR to detect the transcript levels of the DNA methyltransferase genes. We analyzed the relative expression of MTase and found that, in the HN field, the expression of *OsDRM3* and *OsCMT3* was significantly lower in Ox1-LN compared with Ox1-HN and there was no difference in *OsMET1*, *OsDRM2,* and *OsCMT2* (Fig. 8a-e), and the expression of all MTase genes showed no difference between WYG7-LN and WYG7-HN (Fig. 8a-e). However, in the NN field, the expression of *OsDRM2* and *OsCMT2* was significantly lower in WYG7-LN compared with WYG7-HN, and all MTase genes showed no difference between Ox1-LN and Ox1-HN expression (Fig. 8f-j). The results suggested that in the HN condition, LPSN of Ox1 could influence the expression of *OsDRM2* and *OsCMT2*, and in the NN condition, LPSN of WYG7 could influence the expression of *OsDRM3* and *OsCMT3*. Therefore, LPSN can affect the different types of methylation in the Ox1 line under and WYG7 under HN and NN treatments.

### Growth difference induced by parent seed N content is not heritable

We collected seeds of WYG7-LN, WYG7-HN, Ox1-LN, and Ox1-HN from the HN and NN field in S1 generation, and planted eight lines in HN and NN field for S2 generation to analyze if the influence of LPSN on growth is inheritable. We listed characteristics of phenotype for 16 lines in S2 generation in Table S6 and the comparison of WYG7 and Ox1 with different parent seed N content in Table 1. We found that LPSN could influence Ox1 growth in the HN field in S1 generation, but the influence was not inherited to S2 generation. The plant height, grain yield, seed N concentration, seed total N content and plant total N content in Ox1-LN all decreased compared with Ox1-HN in the HN fertilizer field in S1 generation, but none of these phenotypes showed a decrease in S2 generation.

**Table 1:** Phenotype changes influenced by seed N content during two generations

Field N fertilizer			Material	Plant Height	Tiller number	Grain yield	Seed N concentration	Seed total N content	Plant total N content	Relative expression of *OsNAR2.1*
S2		S1								
S1		HN	WYG7-LN vs WYG7-HN	NS	NS	NS	NS	NS	NS	NS
		NN	WYG7-LN vs WYG7-HN	NS	NS	NS	NS	NS	NS	NS
		HN	Ox1-LN vs Ox1-HN	↓	NS	↓	↓	↓	↓	↓
		NN	Ox1-LN vs Ox1-HN	NS	NS	NS	NS	NS	NS	NS
S2	HN	HN	WYG7-LN vs WYG7-HN	NS	NS	NS	NS	NS	NS	NS
		NN	WYG7-LN vs WYG7-HN	NS	NS	NS	NS	NS	NS	NS
		HN	Ox1-LN vs Ox1-HN	NS	↑	NS	NS	NS	NS	NS
		NN	Ox1-LN vs Ox1-HN	NS	↑	NS	NS	NS	NS	NS
	NN	HN	WYG7-LN vs WYG7-HN	NS	NS	NS	NS	NS	NS	NS
		NN	WYG7-LN vs WYG7-HN	NS	NS	NS	NS	NS	NS	NS
		HN	Ox1-LN vs Ox1-HN	NS	NS	NS	NS	NS	NS	NS
		NN	Ox1-LN vs Ox1-HN	NS	NS	NS	NS	NS	NS	NS

NS means no significant difference. Arrowhead indicates upregulate and downregulate, red indicates upregulate and green indicates downregulate

## Discussion

Nitrogen deficiency is severe environmental stress which impairs crop production and prevents plants from effectively absorbing enough nitrogen for growth [4]. There are many reports where environmental nitrogen stress affects the growth of rice, including a decrease in the yield and seed setting rate [2, 5, 39]. In our research, the stress of nitrogen deficiency caused the decrease of plant height, and grain yield, seed setting rate, and seed N content of WYG7 and the high N-use efficiency line Ox1. However, for WYG7, nitrogen deficiency-induced phenotypic changes but did not affect the expression of *OsNAR2.1*. In Ox1 line, nitrogen deficiency not only affected its phenotype but also induced a decrease in the expression of *OsNAR2.1* (Fig. 1, Table S1). Furthermore, the decrease in the growth, grain yield, and *OsNAR2.1* expression was inherited in the second generation despite the restoration of high nitrogen supply conditions in the Ox1 line, rather than WYG7. This decrease, however, did not persist in the third generation.

To confirm that the LPSN could influence the growth of *OsNAR2.1* overexpression line, we also grown overexpression *OsNAR2.1* line (Ov199) and *OsNAR2.1* RNAi knock-down line (RNAi) of Nippobare background with wild type (NP) [25, 26] with difference seed N content in four different N content field. The results showed that the plant height, grain yield, total N content of seeds and total N content of plant significantly decrease 22.3, 33, 50 and 38%, respectively, in Ov199-LN compared with Ov199-HN in HN field (Fig. S10 and S11), which is similar with Wuyungeng7 background. And the expression of *OsNAR2.1* only half in Ov199-LN compared with Ov199-HN (Fig. S12). But the plant height, grain yield, total N content of seeds, total N content and the expression of *OsNAR2.1* in NP not influenced by parent seed N content, also similar in Wuyungeng7. Same results in RNAi line (Fig. S10–12). The results suggested that LPSN could induce the decrease of the expression of *OsNAR2.1* and reduce the growth of *OsNAR2.1* overexpression lines in both Wuyungeng7 and Nippobare backgrounds.

Since there have been reports suggesting that the reduced nitrogen content in seeds is endogenous nitrogen stress for plants, which also could affect plant growth [9], we considered that field N deficiency would decrease the seed N content in the Ox1 line and influence the growth in the S2 generation. It has been previously reported that higher seed nutrition was associated with increased seedling growth [9, 40], but not in all plants [9–11, 41]. The difference in seed nitrogen content does not influence the seedling growth in wheat [41], but influences soybean seedling fertility [9], could improve the salinity tolerance in wheat seedlings, and enhances seed vigor and accelerates germination time in rice [10, 11]. We generated seeds with significant different N content of WYG7 and Ox1 in water with and without nitrogen to analyze the growth of seedling response to different N fertilizer in the water. Our results showed that in both N deficiency and N fertilizer situations, LPSN retarded the seedling growth on the Ox1 line but had no influence on WYG7 (Figs. 2 and 3). The relative expression of *OsNAR2.1* was significantly lower in LPSN Ox1 seedlings in N fertilizer condition, but not in N deficiency condition (Fig. 3i, j), with no differences between WYG7-LN and WYG7-HN seedlings in both conditions (Fig. 3i-l). We concluded that at the seedling stage, LPSN Ox1 line retarded seedling growth and decreased expression of *OsNAR2.1* in water with external N, but not in WYG7.

Some previous studies suggest that early growth guarantees higher biomass and grain yield under optimal management conditions [42, 43], while others report that early vigorous growth does not ensure a higher yield at maturity [44]. Mayamulla et al. (2017) reported that dry weight of rice in low-fertility field conditions correlated well with seed phosphorus content but less with seed N concentration [44]. In our study, HPSN led to better seedling growth and resulted in higher grain yield and N content in Ox1 under the N fertilizer condition. However, in the N fertilizer deficiency or not optimal management conditions, the growth and yield of mature Ox1 line showed no correlation with early seedling growth.

In our results, LPSN could lead to a decrease in the expression of *OsNAR2.1.* The OsNRT2 family proteins OsNRT2.1, OsNRT2.2, and OsNRT2.3a require the participation of OsNAR2.1 when participating in the absorption of nitrate [26, 29]. Expression of the NRTs is regulated by nitrate, N metabolites, N starvation, circadian rhythm, sucrose, and pH [1]. Higher expression of the NRT2 family results in higher yield in rice [26, 27, 45]. Chen et al. (2017) demonstrated that compared with the wild type, the expression of *OsNRT2.1*, *OsNRT2.2,* and *OsNRT2.3a* genes of the pOsNAR2.1:OsNAR2.1 transgenic line increased by about 73, 68, and 76% in 1.25 mM NH_4_NO_3_. We found that in both +N condition in seedling and HN condition in the field, LPSN induced lower expression of *OsNRT2.1* and *OsNRT2.3a* in Ox1, compared with HPSN (Fig. 5). But by visualization the methylation status of *OsNAR2.1* and *OsNRT*s, we found there is no methylation change induce by LPSN, which indicated the expression changes wasn’t regulated by gene methylation (Fig. S9).

We concluded that LPSN in high N efficiency line Ox1 could lead to reduced seedling growth and resulted in decreased *OsNAR2.1*, *OsNRT2.1,* and *OsNRT2.3*a expression in the field with sufficient nitrogen, further decreasing the grain yield of high N efficiency lines Ox1.

Since the genome does not change in plants during a single generation, we hypothesized that epigenetic changes in a generation influence the phenotype of Ox1 line. Cytosine methylation is responsive to various biotic and abiotic stresses and may produce changes in gene expression and resulting phenotypes [14, 46]. Villalobos et al. (2015) reported that an extensive remodeling of global DNA methylation occurs in *Arabidopsis* plants exposed to low Pi availability, related to changes in gene expression [23]. Kou et al. (2011) reported that N deficiency induces changes in DNA methylation and that 50% of the altered methylation patterns in somatic cells of the first generation of stressed plants were recaptured in the second generation, and were then stably inherited to the third and four generations, providing enhanced tolerance to stress for the offspring [19]. Kou et al. (2011) research focuses on the offspring locus-specific methylation alteration response to environmental N stress in seedling, but our research was more focused on the methylation changes in response to parent seed N deficiency in Ox1 and wild type plants in the field. In our research, we found in HN condition, LPSN caused global methylation level increase in WYG7, and in mCG, mCHG, and mCHH levels (Fig. 6a and Fig. S5a-c). The methylation level change of mCG, mCHG and mCHH was not consistent with mC change in Ox1. Furthermore, we found that the mC level of WYG7-HN and Ox1-HN was similar (17.67 and 17.7%) (Fig. 6a). Previous research shows that in the seedling stage, the relative mC count of WYG7 is significantly higher than of Ox1 [25]. However, in the N deficiency condition, the global methylation level of Ox1 (−HN and -LN) were both higher than WYG7 (−HN and -LN), suggesting that the N deficiency could induce methylation level increase in Ox1 lines. Furthermore, we analyzed the gene element methylation level in WYG7 and Ox1 lines. Previously published results show that DNA methylation at promoters and transposable element (TEs) generally correlates with transcriptional repression [47], the exons tend to be more highly methylated than introns, and the end of the gene shows a similar drop in methylation to the promoter region [48]. Similar to our results, all WYG7 and Ox1 lines had a decrease from promoter to gene and the intron. Previous studies in plants suggested that DNA methylation is found in the CG context of active genes [49]. In our results, the methylation in active genes mostly focused on CG. The CG methylation level was higher in WYG7 lines inactive gene and 5’UTR regions than in Ox1 in N deficiency condition. There are also reports showing the enrichment of DMRs in the intron and intergenic regions, and promoters with conserved and functional regulation of transcription [50, 51]. We found the total methylation level was higher in Ox1 lines in promoter and intergenic regions (Fig. 6e, f) in N deficiency condition. However, in the N fertilizer treatment, the methylation level in different regions showed no difference between four samples (Fig. 6b, c). We hypothesize that in Ox1 line, the influence of field N deficiency is stronger than parent seed N content influence on total methylation level of plants, and DNA methylation in non-coding regions may play a crucial role on responsive gene regulation under N starvation conditions. Furthermore, N deficiency influence on methylation of WYG7 focused on CG context in gene body and 5’UTR region, may causing strong methylation influence on the active genes. Promoters proximal to transcription start sites (TSS) are frequently considered sufficient for the initiation and elongation of transcription, but the level of promoter-driven expression is generally low, and high level of gene expression requires the participation of enhancers [38, 52]. Therefore, we analyzed the methylation level of enhancer [53] and the results show that the LPSN influence on enhancer is stronger in Ox1 under NN condition (Fig. S5 d, f).

The DMRs and DMGs between lines with different seed N content were identified. Villalobos et al. (2015) reported that P deficiency could induce 20% hyper-DMRs in the shoot and 86% hyper-DMRs in the root, and gene ontology enrichment analysis presenting differential methylation in response to low phosphorus was strongly enriched in signaling components [23]. In our research, the DMRs were identified between LPSN and HPSN lines. We found that in the N fertilizer condition, LPSN induced more hypo-DMR in WYG7 and more hyper-DMRs in Ox1. In N deficiency treatment, LPSN induced more hyper-DMR in WYG7 and more hypo-DMRs in Ox1, which suggested that the DNA methylation in response to LPSN was sensitive to external N content. Moreover, in WYG7, LPSN induced more DMGs in the N deficiency field, but in Ox1, induced more DMGs in the N fertilizer field (Fig. 7). The results suggest that, in the N deficiency field, DNA methylation was more sensitive to LPSN in wild type, but in the N fertilizer field, DNA methylation was more sensitive to LPSN in Ox1 line.

To further investigate the methylation influence on the gene patterns, we cluster-analyzed the unique DMGs influence in different N fields and found the number of unique methylation genes in WYG7 and Ox1 in HN condition was similar (4831 and 4617) (Fig. 7c). However, there were 22 clusters significantly enriched by unique Ox1-DMG only and two clusters significantly enriched by unique WYG7-DMG only. However, in NN treatment, LPSN induced more DMGs in WYG7 than in Ox1 and there were 11 clusters significantly enriched by unique WYG7-DMG only (Fig. 7g, h). It is well known that gene methylation is highly correlated with transcription levels [31]. We chose 12 genes that relative to nutrition regulation in rice with DMGs in different lines under HN and NN conditions, the expression of 7 genes upregulated by hypo-methylation (Fig. S7, S8). Zilberman et al. (2007) [52] reported that transcripts from genes normally methylated within the transcribed region are upregulated by loss of methylation, and similar pattern showed in our results.

Since methylation changes in response to LPSN and nitrogen external supply, we proposed that the DNA MTase could be differentially regulated. Greco et al. (2019) reported that cadmium and copper both could induce the upregulation of the DNA methyltransferases *CMT3* and *DRM2* in seagrass [54]. Villalobos et al. (2015) reported that the transcript level of DNA MTases (*MET1*, *DRM1*, *DRM2,* and *CMT3*) is regulated by low phosphorus stress [23]. We found that, compared with HPSN, the expression of *OsDRM2* and *OsCMT2* was significantly reduced in the LPSN Ox1 line. In the N deficiency field, the expression of *OsDRM3* and *OsCMT3* was significantly reduced in the LPSN WYG7 line. DRM2 is responsible for de novo methylation at all sites [55, 56] and CMT2 has a clear-cut preference for asymmetric CHH sites [57]. DRM3 is likely to target- or chromatin context-dependent, and most likely functions exclusively with DRM2 in RNA-directed DNA methylation [58]. CMT3 is plant-specific DNA methyltransferase maintenance methylation of symmetric CHG sites and participates in CHH site methylation [55, 59, 60]. Our results suggest one of the reasons of methylation changes in Ox1 and WYG7 were regulated by MTase. In addition, small RNAs, including microRNAs (miRNAs) and small interfering RNAs (siRNAs), provide RNA-mediated regulation of genome stability and influence gene expression [38, 61]. DNA methylation also can be induced by double-stranded RNA through the RNA interference (RNAi) pathway [61]. It is possible that DNA methylation changes induce by LPSN was associated with RNA-directed methylation during post transcriptional gene silencing (PTGS) [62, 63].

Epigenetic regulation is mediated by differences in DNA methylation at cytosine residues and by post-translational histone modifications [63], and histone modification also correlated with gene activity [64]. Report showed that repression of NRT2.1 transcription by high N supply is associated with an HNI9/AtIWS1-dependent increase in histone H3 lysine 27 trimethylation at the NRT2.1 locus [16]. OsNAR2.1 as pattern protein with OsNRT2.1 could also influenced by histone modification. Therefore, the methylation change is one of the reasons that LPSN induce growth difference in Ox1. Other epigenetic regulation, like histone modification, could also be a part of the process.

Previous studies report the inheritance of environmentally induced epigenetic variations [65], which can cause heritable phenotypic modifications [66]. Kou et al. (2010) reported that N-deficiency could induce locus-specific alteration in cytosine methylation patterns in rice (S0) and the altered methylation patterns are detected at frequencies ranging from 47 to 59% in the filial rice (S1) [19]. They found that two groups of S2 plants descended from the same N-deficiency-stressed S0-derived S1 individual. One inherited the stress-modified methylation patterns and phenotypes acquired at S0. In order to determine if the phenotype changes caused by LPSN were heritable in our research, we planted eight lines of WYG7-LN, WYG7-HN, Ox1-LN, and Ox1-HN from HN and NN fields for another year, and found the phenotype changes induced by LPSN in Ox1 were non-heritable. Therefore, we conclude that not all the methylation changes induced by LPSN were heritable and the heritable changes were not strong enough to influence the phenotypes.

We hypothesize that in the N deficiency field, LPSN caused a significant number of gene methylation changes, but the expression of *OsNAR2.1* in both WYG7 and Ox1 lines could not be influenced. Therefore, the phenotype of both wild type and high N-use efficiency Ox1 lines was strongly influenced by the external N deficiency, overcoming the influence of parent seed N content. In the HN field, the parent seed nitrogen content decreases induced DNA methylation changes at the epigenetic level and significantly decreased the expression of *OsNAR2.1*, resulting in a heritable phenotype of N deficiency over two generations in the Ox1 line.

## Conclusion

In our study, we found that the parent seed N content decrease in Ox1 could retard filial seedling growth. Furthermore, induce a decrease in filial seed N content, plant N content, and grain yield in mature with high external N supply. However, the growth of WYG7 in both seedling and mature are not influenced by LPSN. To determine the mechanism underlying the phenotype, we sequenced the methylation data of Ox1 and WYG7 lines with different parent seed N content in different external N. The results demonstrated that LPSN caused massive gene methylation changes and differential methylation gene enriched in over 20 GO pathways in filial Ox1. Furthermore, the expression of *OsNAR2.1* in LPSN filial Ox1 was significantly reduced compared to HPSN filial plants in high external N, but the expression of *OsNAR2.1* in LPSN filial wild type did not change in high or no external N. Therefore, we concluded that the parent seed nitrogen content decreases induced DNA methylation changes at the epigenetic level and significantly decreased the expression of *OsNAR2.1*, resulting in a heritable phenotype of N deficiency over two generations in the overexpression line. Our results maybe point out an innovative view in plant growth responds to endogenous nitrogen stress and indicate a new direction in breeding of high nutrient efficiency plants.

## Materials and methods

### Plant materials and growth conditions

*OsNAR2.1* overexpression line (Ox1) with background of *Oryza sativa* L. ssp. *japonica* cv. Wuyugeng7 (WYG7) has been described in Chen et al. (2017) [27]. *OsNAR2.1* overexpression line (Ov199) and RNAi line (RNAi) with background *Oryza sativa* L. ssp. *japonica* cv. Nippobare (NP) has been described in Yan et al. (2011) [26] and Fan et al., (2020) [25]. For plants growing in the field, all materials were grown in plots in the Agricultural Experiment Station of Zhejiang University, Changxing, Zhejiang. Changxing is located in a subtropical monsoon climate zone. The pH of the soil is 7.2. Potassium and phosphate were added at standard levels in the fields (225 and 450 kg/ha). Plants were planted in the field fertilized at a rate of 300 kg N/ha as HN field, 150 N/ha as MN field, 75 N/ha as LN field, and no N fertilizer as NN field. Plots size was 2 × 2.5 m and the seedlings were planted in 10 × 10 arrays [67, 68]. We randomly chose five seedlings from each plot, avoiding those on the edges.

For plants grown in seedling with hydroponics, all seeds collected from T5 were surface-sterilized with 10% (*v*/*v*) hydrogen peroxide solution for 30 min, thoroughly rinsed, and washed six times with deionized water. Seeds of uniform size were germinated on the top cover of a 1 L pot with 40 holes per top cover and one seed per hole. Plants were grown in the greenhouse under natural light at day/night temperatures of 30 °C/22 °C and 60% relative humidity. The containers were filled with water with with 2.5 mM of NH_4_NO_3_ or pure water. Ten-day-old seedlings were transplanted into holes on the top cover of a 7 L pot.

### Biomass and total nitrogen (N) measurement

We harvested WYG7 and Ox1 lines from the field and heated them at 105 °C for 30 min, and then dried at 75 °C for 3 days. The N concentration and total N content were measured using the Kjeldahl method [30].

For plants to grow with hydroponics, the length of the shoot was tested every 3 days. We harvested WYG7 and Ox1 after 30 days and heated at 105 °C for 30 min. The biomass was estimated as the total dry weight of shoot or root weights.

### Gene expression analysis

Total RNA was extracted from flag leaf tissue using TRIzol reagent (Vazyme Biotech Co, Ltd.). DNase I-treated total RNAs were subjected to reverse transcription (RT) with HiScript II Q Select RT SuperMix for qPCR kit (Vazyme Biotech Co, Ltd.) according to the manufacturer’s instructions. Quantitative assays were performed using AceQTM qPCR SYBR Green Master Mix (Vazyme Biotech Co, Ltd.). The relative expression level was normalized to the amount of *OsActin* (*LOC_Os03g50885*) in the same sample and presented as 2-△CT. All primers used for RT-qPCR are listed in Supplementary Table S7.

### WGBS and data analyses

Genomic DNA was extracted from leaves of mature plants and shipped to the Anoroad Genome Company (Beijing) for Whole Genome Bisulfite Sequencing (WGBS) library preparation and sequencing.

The raw reads were filtered and trimmed to obtain clean reads and the available data were compared with the reference genome of Oryza_sativa Japonica IRGSP-1.0 to obtain the alignment results using Bismark (v0.9.0) [69]. For each C site, the methylation level (%) was calculated with the following formula: 100* (reads supported methylation)/(total reads depth of this site). For the methylated regions, methylation level (%) was calculated by the 100* (methylation level of all C sites in this region)/(total number of C sites in this region).

The differential analysis of methylation was performed at the region-based level, differentially methylated regions (DMRs) [25]. Aberrant DNA methylation was compared with the control group. An increased methylation pattern was defined as hyper-methylation, whereas a decreased methylation pattern was defined as hypo-methylation. DMR detection was performed using SMART (http://fame.edbc.org/smart /readme.html) with: (1) the read coverage of each C > 4; (2) The total number of C in each DMR > = 5; (3) The length of DMR > = 100 bp; (4) The difference of methylation level between groups was greater than or equal to 0.3; (5) The CPG site with *p*-value less than 1e-10 was reserved as the final DMR. Based on the results of DMR genome annotation, genes with overlapping promoter (1Kb upstream) and gene body region consider as differential methylation gene (DMG).

GO analysis was performed using AgriGO (http://systemsbiology.cau.edu.cn/agriGOv2/index.php).

### Statistical analysis

The data were analyzed by Duncan one-way analysis of variance (ANOVA). Statistically significant differences at *P* < 0.05 between samples were indicated by different letters on the histograms or after mean values. All statistical evaluations were conducted using the IBM SPSS Statistics version 23 software (SPSS Inc., Chicago, IL, USA).

## Supplementary Information

**Additional file 1 Table S1** Comparison of agronomic traits of WYG7 and Ox1 in +N and -N fields in S0 generation.. **Table S2** Comparison of agronomic traits of WYG7 and Ox1 in S1 and S2 generation.. **Table S3** Agronomic traits of WYG7 and Ox1 lines with different parent seed N content in the different N field. **Table S4** Agronomic traits of WYG7 and Ox1 lines with different parent seed N content in the different N field in S2. **Table S5** Samples sequencing data in this study. **Table S6** Characteristics of phenotype for Ox1 and WYG7 with different seed N content for two-year different N fertilizer. **Table S7** Primers for qRT-PCR. **Fig. S1** Parent seed nitrogen content decrease influence on the expression of *OsNRT*s genes. **Fig. S2** The decrease in the parent seed nitrogen content influence on phenotype. **Fig. S3** Plant height and grain yield induce by N deficiency in S0 and S1 generation.. **Fig. S4** The decrease in the parent seed nitrogen content influence on chorophyII content and seed N concentration. **Fig. S5** Methylation level of Ox1 and WYG7 lines. **Fig. S6** Differential methylation induces by field N deficiency in WYG7-HN and Ox1-HN. **Fig. S7** Methylation status and gene expression of relative genes in HN field. **Fig. S8** Methylation status and gene expression of relative genes in NN field. **Fig. S9** Methylation status of OsNRT family genes in different N field. **Fig. S10** The decrease in parent seed nitrogen content of Ov199 leads to plant height and grain yield per plant decrease. **Fig. S11** The decrease in parent seed nitrogen content of Ov199 leads to total N content of seeds and plant decrease. **Fig. S12** The decrease in parent seed nitrogen content of Ov199 leads to relative expression of *OsNAR2.1* decrease. The decrease in parent seed nitrogen content of Ov199 leads to relative expression of *OsNAR2.1* decrease. (A-D) Relative expression of *OsNAR2.1 *of different lines in the field with HN, MN, LN and NN fertilizer. Error bars: SD (*n* = 4). Significant differences between different lines are indicated by different letters (*P* < 0.05, one-way ANOVA, Duncan).

## Data Availability

The Whole Genome Bisulfite Sequencing (WGBS) datasets (in Materials and Methods: WGBS and Data Analyses) generated during the current study are available in the NCBI GEO repository with accession number GSE160884 (https://www.ncbi. nlm.nih.gov/geo/query/acc.cgiacc=GSE160884).
