# Supplementary material for: Plant DNA methylation is sensitive to parent seed N content and influences the growth of rice
Source: BMC Plant Biol. 2021 May 11;21:211. doi: 10.1186/s12870-021-02953-3 (PMC8111971; doi:10.1186/s12870-021-02953-3)
Supplement: Supplementary file 1 — Additional file 1 Table S1 Comparison of agronomic traits of WYG7 and Ox1 in +N and -N fields in S0 generation.. Table S2 Comparison of agronomic traits of WYG7 and Ox1 in S1 and S2 generation.. Table S3 Agronomic traits of WYG7 and Ox1 lines with different parent seed N content in the different N field. Table S4 Agronomic traits of WYG7 and Ox1 lines with different parent seed N content in the different N field in S2. Table S5 Samples sequencing data in this study. Table S6 Characteristics of phenotype for Ox1 and WYG7 with different seed N content for two-year different N fertilizer. Table S7 Primers for qRT-PCR. Fig. S1 Parent seed nitrogen content decrease influence on the expression of OsNRTs genes. Fig. S2 The decrease in the parent seed nitrogen content influence on phenotype. Fig. S3 Plant height and grain yield induce by N deficiency in S0 and S1 generation.. Fig. S4 The decrease in the parent seed nitrogen content influence on chorophyII content and seed N concentration. Fig. S5 Methylation level of Ox1 and WYG7 lines. Fig. S6 Differential methylation induces by field N deficiency in WYG7-HN and Ox1-HN. Fig. S7 Methylation status and gene expression of relative genes in HN field. Fig. S8 Methylation status and gene expression of relative genes in NN field. Fig. S9 Methylation status of OsNRT family genes in different N field. Fig. S10 The decrease in parent seed nitrogen content of Ov199 leads to plant height and grain yield per plant decrease. Fig. S11 The decrease in parent seed nitrogen content of Ov199 leads to total N content of seeds and plant decrease. Fig. S12 The decrease in parent seed nitrogen content of Ov199 leads to relative expression of OsNAR2.1 decrease. The decrease in parent seed nitrogen content of Ov199 leads to relative expression of OsNAR2.1 decrease. (A-D) Relative expression of OsNAR2.1 of different lines in the field with HN, MN, LN and NN fertilizer. Error bars: SD (n = 4). Significant differences between different lines are indi [file 12870_2021_2953_MOESM1_ESM.pdf]

# Plant DNA methylation is sensitive to parent seed N content and influences the growth of rice

Xiaoru Fan[1], Laihua Liu[4], Kaiyun Qian[1], Jingguang Chen[1,2], Yuyue Zhang[1], Peng Xie[1], Man Xu[1], Zhi Hu[1], WenKai Yan[3], Yufeng Wu[3], Guohua Xu[1] and Xiaorong Fan[1*]

[1] State Key Laboratory of Crop Genetics and Germplasm Enhancement, MOA Key Laboratory of Plant Nutrition and Fertilization in Low-Middle Reaches of the Yangtze River, Nanjing Agricultural University, 210095 Nanjing, China

[2] School of Agriculture, Sun Yat-sen University, Guangzhou, Guangdong 510275, PR China

[3] Bioinformatics Center, Nanjing Agricultural University, 210095 Nanjing, China

4 Vazyme Biotech Co., Ltd, 210033, Nanjing, China

***** Correspondence: xiaorongfan@njau.edu.cn; Tel/Fax: 0086-025-84396238

† Present Address: Xiaorong Fan, College of Resource and Environmental Science, Nanjing Agricultural University, Nanjing 210095, China

Supporting information: 12 figures and 7 tables

## Supporting Information

## Table S1 Agronomic traits of WYG7 and Ox1 in +N and -N fields in S0 generation.

| | N treatment | Panicle length(cm) | Grain weight (g/panicle) | Seed setting rate (%) | Grain number per panicle | 1000-grain weight(g) | Length of seeds | Wide of seeds |
|---|---|---|---|---|---|---|---|---|
| WYG7 | S0+N | 17.70±1.12a | 3.17±0.41a | 0.95±0.03a | 129.80±16.87ab | 25.83±1.58a | 7.61±0.11a | 3.34±0.12ab |
| | S0-N | 13.98±0.79b | 2.76±0.16ab | 0.88±0.03b | 114.20±12.60b | 27.77±0.63a | 7.51±0.23a | 3.40±0.10a |
| Ox1 | S0+N | 17.52±0.65a | 2.88±0.27ab | 0.95±0.04a | 121.20±7.26a | 25.89±2.10a | 7.56±0.14a | 3.30±0.08b |
| | S0-N | 15.04±1.38b | 2.47±0.21b | 0.78±0.05c | 105.80±12.99ab | 26.96±1.13a | 7.56±0.23a | 3.30±0.14b |

Note: Error bars: SD (n = 10 plants). Significant differences between different lines are indicated by different letters ($P < 0.05$, one-way ANOVA).

## Table S2 Agronomic traits of WYG7 and Ox1 in S1 and S2 generation.

| | | | S1 N treatment | $S_0$ N treatment | Panicle length (cm) | Grain weight (g/panicle) | Seed setting rate (%) | Grain number per panicle | 1000-grain weight (g) |
|---|---|---|---|---|---|---|---|---|---|
| S1 | WYG7 | | S1+N | $S_0$+N | 17.15±0.67a | 3.43±0.37a | 0.73±0.04a | 164.25±26.78a | 19.82±2.75a |
| | | | | $S_0$-N | 16.58±0.57ab | 3.65±0.25a | 0.70±0.05ab | 177.60±16.68a | 21.45±1.66a |
| | Ox1 | | | $S_0$+N | 17.10±1.43a | 3.40±0.39a | 0.75±0.02a | 152.00±22.58ab | 21.36±1.46a |
| | | | | $S_0$-N | 15.80±1.21b | 2.46±0.36b | 0.67±0.02b | 129.00±20.11b | 22.34±2.22a |
| S2 | WYG7 | S2+N | S1+N | $S_0$+N | 17.18±0.42b | 3.62±0.48a | 0.95±0.01a | 136.00±16.29a | 27.46±1.07a |
| | | | | $S_0$-N | 16.46±1.31ab | 2.98±0.28a | 0.94±0.02a | 118.60±9.92a | 26.42±0.82a |
| | Ox1 | | | $S_0$+N | 17.80±0.86a | 3.58±0.38a | 0.90±0.07a | 142.20±16.68a | 27.67±1.46a |
| | | | | $S_0$-N | 18.06±0.30a | 3.11±0.20a | 0.91±0.02a | 135.00±21.62a | 26.86±1.30a |

Note: Error bars: SD (n = 10 plants). Significant differences between different lines are indicated by different letters ($P < 0.05$, one-way ANOVA).

**Table S3 Agronomic traits of WYG7 and Ox1 lines with different parent seed N content in the different N field**

| Field N fertilizer | Material | Panicle length (cm) | Grain weight (g/panicle) | Seed setting rate (%) | Grain number per panicle | 1000-grain weight (g) |
|---|---|---|---|---|---|---|
| HN | WYG7-HN | 17.15±0.67a | 3.43±0.38a | 0.73±0.04a | 164.25±26.78a | 19.82±2.75a |
| | WYG7-LN | 16.58±0.57ab | 3.65±0.25a | 0.70±0.05ab | 177.60±16.68a | 21.45±1.66a |
| | Ox1-HN | 17.10±1.43a | 3.40±0.39a | 0.75±0.02a | 152.0±22.58ab | 21.36±1.46a |
| | Ox1-LN | 15.80±1.21b | 2.460±0.36b | 0.67±0.02b | 129.00±20.11b | 22.34±2.21a |
| MN | WYG7-HN | 15.98±0.98a | 2.93±0.48a | 0.71±0.04a | 155.00±24.71a | 23.08±0.61a |
| | WYG7-LN | 17.05±0.85a | 2.87±0.24a | 0.67±0.06a | 140.57±9.61a | 22.44±2.36ab |
| | Ox1-HN | 16.58±0.91a | 2.71±0.36a | 0.71±0.06a | 145.00±10.61a | 22.63±1.02ab |
| | Ox1-LN | 16.28±0.89a | 3.17±0.36a | 0.72±0.06a | 148.00±26.00a | 20.98±0.80b |
| LN | WYG7-HN | 15.26±1.10a | 3.07±0.47a | 0.78±0.05a | 150.40±27.15a | 22.07±1.68ab |
| | WYG7-LN | 15.80±1.11a | 3.18±0.62a | 0.80±0.04a | 144.80±29.78a | 20.22±0.95b |
| | Ox1-HN | 15.56±1.45a | 2.58±0.55a | 0.80±0.07a | 120.00±32.16a | 20.62±1.38b |
| | Ox1-LN | 15.18±0.90a | 2.68±0.32a | 0.70±0.24a | 159.80±92.62a | 22.92±1.68a |
| NN | WYG7-HN | 15.30±0.49a | 3.10±0.45a | 0.76±0.08a | 139.00±14.41a | 22.51±1.01a |
| | WYG7-LN | 16.14±0.83a | 3.22±0.92a | 0.80±0.05a | 148.60±14.48a | 23.63±1.27a |
| | Ox1-HN | 15.68±0.38a | 2.73±0.33a | 0.80±0.05a | 130.25±12.71a | 21.47±2.58a |
| | Ox1-LN | 15.36±0.71a | 2.86±0.34a | 0.77±0.04a | 135.00±7.75a | 22.63±1.82a |

Note: Error bars: SD (n = 10 plants). Significant differences between different lines are indicated by different letters ($P < 0.05$, one-way ANOVA).

## Table S4 Agronomic traits of WYG7 and Ox1 lines with different parent seed N content in the different N field in S2

| S2 | | | | | | | | |
|---|---|---|---|---|---|---|---|---|
| Field N fertilizer | Material | Plant height （cm） | Tillering number | Grain yield per plant(g) | Seed N concentration （mg/g） | Seed total N content （mg/plant） | Plant total N content （mg/plant） | Relative expression of *OsNAR2.1* |
| HN | WYG7-HN | 89.88±4.82a | 7.29±0.76a | 18.67±0.56b | 10.00±1.93ab | 201.48±44.57bc | 201.48±44.57bc | 0.49±0.2c |
| | WYG7-LN | 88.70±2.98ab | 7.25±0.89a | 20.07±5.02b | 9.91±2.29ab | 226.96±40.97ab | 226.96±40.97ab | 0.20±0.07c |
| | Ox1-HN | 92.20±3.11a | 7.00±1.31a | 25.28±2.74a | 12.26±2.10a | 273.64±18.66a | 273.64±18.66a | 7.344±1.442a |
| | Ox1-LN | 85.70±2.79b | 7.10±0.88a | 18.80±3.17b | 8.55±0.72b | 166.21±40.75c | 166.21±40.75c | 3.962±0.538b |
| MN | WYG7-HN | 86.50±6.12a | 5.80±0.92b | 21.12±4.60a | 9.61±1.94a | 182.39±28.56a | 182.39±28.56a | 0.61±0.26b |
| | WYG7-LN | 84.50±3.44a | 5.86±1.46b | 20.65±3.00a | 8.11±1.02a | 165.15±7.50a | 165.15±7.50a | 0.57±0.28b |
| | Ox1-HN | 88.56±3.28a | 7.89±0.93a | 18.28±3.35a | 8.58±1.38a | 151.36±26.93a | 151.36326.93a | 9.70±5.09a |
| | Ox1-LN | 85.67±3.67a | 7.63±1.41a | 21.05±2.74a | 7.71±1.32a | 161.56±31.16a | 161.58±31.16a | 7.71±2.42a |
| LN | WYG7-HN | 87.29±1.89b | 6.67±1.86a | 22.35±3.71a | 7.92±1.11a | 158.32±28.75a | 158.32±28.75a | 0.20±0.07b |
| | WYG7-LN | 86.10±2.33b | 6.29±1.11a | 19.11±5.42a | 7.68±0.17a | 133.08±15.86a | 133.08±15.86a | 0.21±0.04b |
| | Ox1-HN | 89.06±2.08a | 7.13±1.73a | 22.63±4.17a | 6.67±2.33ab | 168.38±83.70a | 168.38±83.70a | 9.84±4.98a |
| | Ox1-LN | 90.22±2.17a | 7.13±1.36a | 18.89±0.83a | 5.36±0.87b | 105.37±19.59a | 105.37±19.58a | 7.15±2.24a |
| NN | WYG7-HN | 81.1431.35b | 5.88±1.13a | 15.96±2.94a | 7.74±1.48a | 148.84±32.52a | 148.84±32.52a | 0.38±0.02b |
| | WYG7-LN | 80.33±1.21b | 5.00±1.12a | 13.16±0.90a | 7.93±0.63a | 102.73±6.32a | 102.73±6.32a | 0.36±0.02b |
| | Ox1-HN | 84.17±3.13a | 5.13±0.99a | 13.02±4.42a | 8.32±0.74a | 107.33±35.82a | 107.33±35.82a | 3.652±0.322a |
| | Ox1-LN | 85.33±1.74a | 5.78±1.30a | 15.07±6.41a | 7.27±1.01a | 116.52±75.11a | 116.52±75.11a | 3.612±0.589a |

Note: Error bars: SD (n = 10 plants). Significant differences between different lines are indicated by different letters ($P < 0.05$, one-way ANOVA).

## Table S5 Samples sequencing data in this study

| Field N fertilizer | Material | Clean Reads | Valid C | | | | mC Count/Ratio of sites | | | |
|---|---|---|---|---|---|---|---|---|---|---|
| | | | 5*C | 5*CG | 5*CHG | 5*CHH | mC | mCG/Ratio | mCHG/Ratio | mCHH/Ratio |
| HN | WYG7-HN | 139,722,670 | 108,890,441 | 16,169,100 | 17,324,668 | 75,396,673 | 21,304,873 | 11,584,008/ 54.37% | 6,084,672/ 28.56% | 3,635,002/ 17.06% |
| | WYG7-LN | 133,589,336 | 104,841,041 | 15,449,192 | 16,541,565 | 72,850,284 | 21,517,825 | 11,364,931/ 52.81% | 6,121,821/ 28.45% | 4,029,071/ 18.72% |
| | Ox1-HN | 135,738,670 | 108,284,879 | 16,189,801 | 17,250,212 | 74,844,866 | 20,878,203 | 11,751,396/ 56.28% | 5,964,903/ 28.57% | 3,160,232/ 15.13% |
| | Ox1-LN | 134,660,004 | 107,478,138 | 15,945,926 | 17,075,422 | 74,456,790 | 21,429,444 | 11,473,825/ 53.54% | 5,777,378/ 26.96% | 4,178,090/ 19.49% |
| NN | WYG7-HN | 139,048,002 | 101,310,179 | 14,682,705 | 15,878,323 | 70,749,151 | 19,525,779 | 10,951,088/ 56.08% | 5,539,464/ 28.37% | 3,034,757/ 15.54% |
| | WYG7-LN | 139,516,004 | 102,560,917 | 14,859,497 | 16,147,775 | 71,553,645 | 19,474,986 | 10,830,917/ 55.61% | 5,507,526/ 28.28% | 3,135,240/ 16.09% |
| | Ox1-HN | 136,773,336 | 113,975,216 | 17,619,309 | 18,310,579 | 78,045,328 | 22,234,904 | 11,951,068/ 53.74% | 6,248,008/ 28.10% | 4,035,490/ 18.14% |
| | Ox1-LN | 138,650,670 | 109,802,502 | 16,598,531 | 17,481,738 | 75,722,33 | 23,356,814 | 12,178,677/ 52.14% | 6,472,173/ 27.71 | 4,705,775/ 20.14% |

**Table S6 Characteristics of phenotype for Ox1 and WYG7 with different seed N content for two-year different N fertilizer**

| Field N fertilizer | | Material | Plant Height (cm) | Tillering number | Grain yield (g) | Seed N concentration (mg/g) | Seed total N content (mg/plant) | Plant total N content (mg/plant) | Relative expression of *OsNAR2.1* |
|---|---|---|---|---|---|---|---|---|---|
| S2 | S1 | | | | | | | | |
| HN | HN | WYG7-HN | 89.86±4.82abc | 7.29±0.76b | 18.67±0.56b | 10.00±1.93bc | 201.48±44.57bc | 287.84±65.00abc | 0.49±0.20c |
| | | WYG7-LN | 92.60±4.77ab | 6.90±1.45b | 18.72±4.70b | 9.67±1.21bc | 185.13±71.86c | 208.81±71.31c | 0.37±0.15c |
| | NN | WYG7-HN | 88.70±2.98abc | 7.25±0.89b | 20.08±5.02b | 9.91±2.29bc | 226.96±40.97abc | 329.23±39.87ab | 0.20±0.07c |
| | | WYG7-LN | 89.70±6.99abc | 7.10±0.74b | 18.79±3.64b | 9.63±0.72bc | 182.89±50.50c | 215.73±50.91c | 0.25±0.11c |
| | HN | Ox1-HN | 92.20±3.11b | 7.00±1.31b | 25.28±2.74a | 12.26±2.10ab | 273.64±18.66ab | 360.70±24.16a | 7.34±1.44a |
| | | Ox1-LN | 94.00±7.70a | 9.40±1.58a | 24.90±1.10a | 12.68±2.38a | 283.16±88.36a | 348.46±64.04a | 7.97±1.53a |
| | NN | Ox1-HN | 85.70±2.79c | 7.10±0.88b | 18.80±3.17b | 8.43±0.68c | 166.21±40.75c | 246.50±47.65bc | 3.96±0.54b |
| | | Ox1-LN | 88.00±6.36bc | 9.40±0.97a | 18.04±0.99b | 9.83±2.24bc | 176.88±39.10c | 216.93±39.98c | 3.13±0.48b |
| NN | HN | WYG7-HN | 81.14±1.35ab | 5.88±1.13a | 15.96±2.94a | 7.74±1.48a | 148.84±32.52a | 187.71±20.75a | 0.38±0.22b |
| | | WYG7-LN | 81.70±4.47ab | 4.90±0.99a | 14.03±4.03a | 7.28±1.32a | 104.20±40.89a | 150.10±27.50b | 1.51±0.86b |
| | NN | WYG7-HN | 80.33±1.21b | 5.00±1.12a | 13.16±0.90a | 7.93±0.63a | 102.73±6.32a | 150.70±11.65ab | 0.43±0.37b |
| | | WYG7-LN | 80.80±4.94b | 5.00±1.33a | 15.05±2.93a | 6.62±2.76a | 102.65±52.71a | 167.09±61.65ab | 0.36±0.20b |
| | HN | Ox1-HN | 84.17±3.13ab | 5.13±0.99a | 13.02±4.42a | 8.32±0.74a | 107.33±35.82a | 152.96±41.86ab | 36.52±3.22a |
| | | Ox1-LN | 82.90±4.33ab | 5.80±1.14a | 14.05±3.77a | 7.19±1.18a | 100.12±29.20a | 169.30±26.75ab | 34.27±11.66a |
| | NN | Ox1-HN | 85.33±1.74ab | 5.78±1.30a | 15.07±6.41a | 7.28±1.01a | 116.52±75.11a | 124.80±15.34b | 36.12±5.89a |
| | | Ox1-LN | 82.20±4.21ab | 5.90±1.29a | 12.94±3.27a | 7.69±1.39a | 97.41±22.25a | 145.95±24.28ab | 32.73±2.99a |

Note: Error bars: SD (n = 5 plants) except for Relative expression of OsNAR2.1, which SD (n = 4 plants). Significant differences between different lines are indicated by different letters ($P < 0.05$, one-way ANOVA).

# Table S7 Primers for RT-PCR

| Gene name | Type of PCR | Primer | Sequence |
|---|---|---|---|
| OsNAR2.1 | quantitative real-time | 5’（F） | GTCGTCGAGAAGCGCAAGA |
| LOC_Os02g38230 | PCR | 5’（R） | GTCCACTGAAGCTGCGAACTT |
| OsDRM2 | quantitative real-time | 5’（F） | AAGTTCGAGTGGGACACAGACG |
| LOC_Os03g02010 | PCR | 5’（R） | TTGGCCTTCCCATTTGCATCCTG |
| OsDRM3 | quantitative real-time | 5’（F） | AGGGATGCTGCTGTCAATCCAC |
| LOC_Os05g04330 | PCR | 5’（R） | AAGAGCTTGGCTGTCCTTCCTC |
| OsMET1 | quantitative real-time | 5’（F） | TAGAGGCAGGGGCTTATGGT |
| LOC_Os03g58400 | PCR | 5’（R） | TTGACAGCGGCGTAGAACTT |
| OsCMT2 | quantitative real-time | 5’（F） | AGTGGATGGTGGTGGTATTGTGT |
| LOC_Os05g13780 | PCR | 5’（R） | CTGTGTCTTTGGAGGTTGAGGG |
| OsCMT3 | quantitative real-time | 5’（F） | GAGCAGAGCCACACAACCAG |
| LOC_Os10g01570 | PCR | 5’（R） | GGAGTAACCCAATGCACGAG |
| OsActin | quantitative real-time | 5’（F） | AGGATTACCATGGCCTCAAGAG |
| LOC_Os03g50885 | PCR | 5’（R） | CGCGTATCTTCCCCATGAAG |
| OsMADS37 | quantitative real-time | 5’（F） | CCGGTGCCTAAGATAAACCA |
| LOC_Os08g41960 | PCR | 5’（R） | TCCTCTTCTTCTCGCTTTGG |
| OsMADS95 | quantitative real-time | 5’（F） | GAGGGAGCTCTCCATCCTCT |
| LOC_Os01g23780 | PCR | 5’（R） | GAGCTTCAACTCGAGGCACT |
| OsMADS59 | quantitative real-time | 5’（F） | AAGCACTGGCCGTCTCTATG |
| LOC_Os06g23950 | PCR | 5’（R） | GATCATGTGCCAACAACTGC |
| OsWAKY46 | quantitative real-time | 5’（F） | GAAGCAAGTCCAGCAGAAGG |
| LOC_Os11g02480 | PCR | 5’（R） | AAGAATGTGGGCTGTTCACC |
| OsWAKY54 | quantitative real-time | 5’（F） | GGAAATGGAGCAAGGATTGA |
| LOC_Os05g40080 | PCR | 5’（R） | GACTGCTGGACTTGCTTCGT |
| OsWAKY59 | quantitative real-time | 5’（F） | AAGCAAGTCCAGCAGAAGGA |
| LOC_Os11g02480 | PCR | 5’（R） | AAGAATGTGGGCTGTTCACC |
| OsATL16 | quantitative real-time | 5’（F） | TGCTGATCTCTTCGGTGTTG |
| LOC_Os01g41400 | PCR | 5’（R） | GGTGATCAGGGTCGTTAGGA |
| OsAMT4 | quantitative real-time | 5’（F） | GAACAACGTCCTGCTCACG |
| LOC_Os03g53780 | PCR | 5’（R） | AAGACGAAGCTGTCGAGGAG |
| OsAGO1a | quantitative real-time | 5’（F） | AGGGATGGTGTCAGTGAAGG |
| LOC_Os02g45070 | PCR | 5’（R） | CGCTGGTCCTTGTGGTTATT |
| OsDRM1b | quantitative real-time | 5’（F） | TCACCTGTTCTTCCCATTCC |
| LOC_Os12g01800 | PCR | 5’（R） | CTGCTGGAGGTAGTGCATCA |

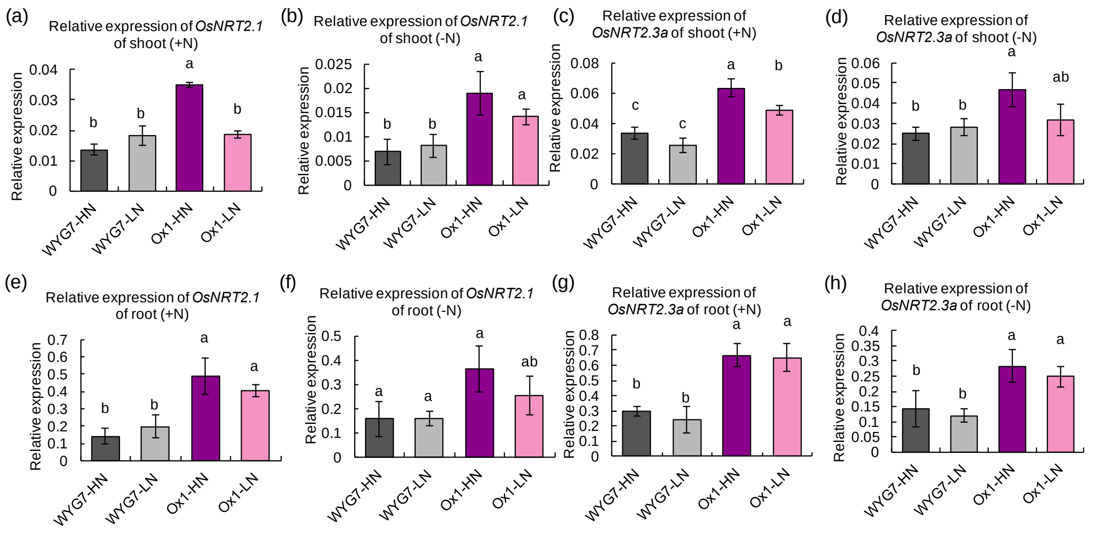


**Fig. S1. Parent seed nitrogen content decrease influence on the expression of *OsNRT*s genes**

(a, b) Relative expression of *OsNRT2.1* of shoot in +N and -N water. Error bars: SD (n = 4 plants). (c, d) Relative expression of *OsNRT2.3a* of shoot in +N and -N water. Error bars: SD (n = 4 plants). (e, f) Relative expression of *OsNRT2.1* of root in +N and -N water. Error bars: SD (n = 4 plants). (c, d) Relative expression of *OsNRT2.3a* of root in +N and -N water. Error bars: SD (n = 4 plants). Significant differences between different lines are indicated by different letters (P < 0.05, one-way ANOVA).

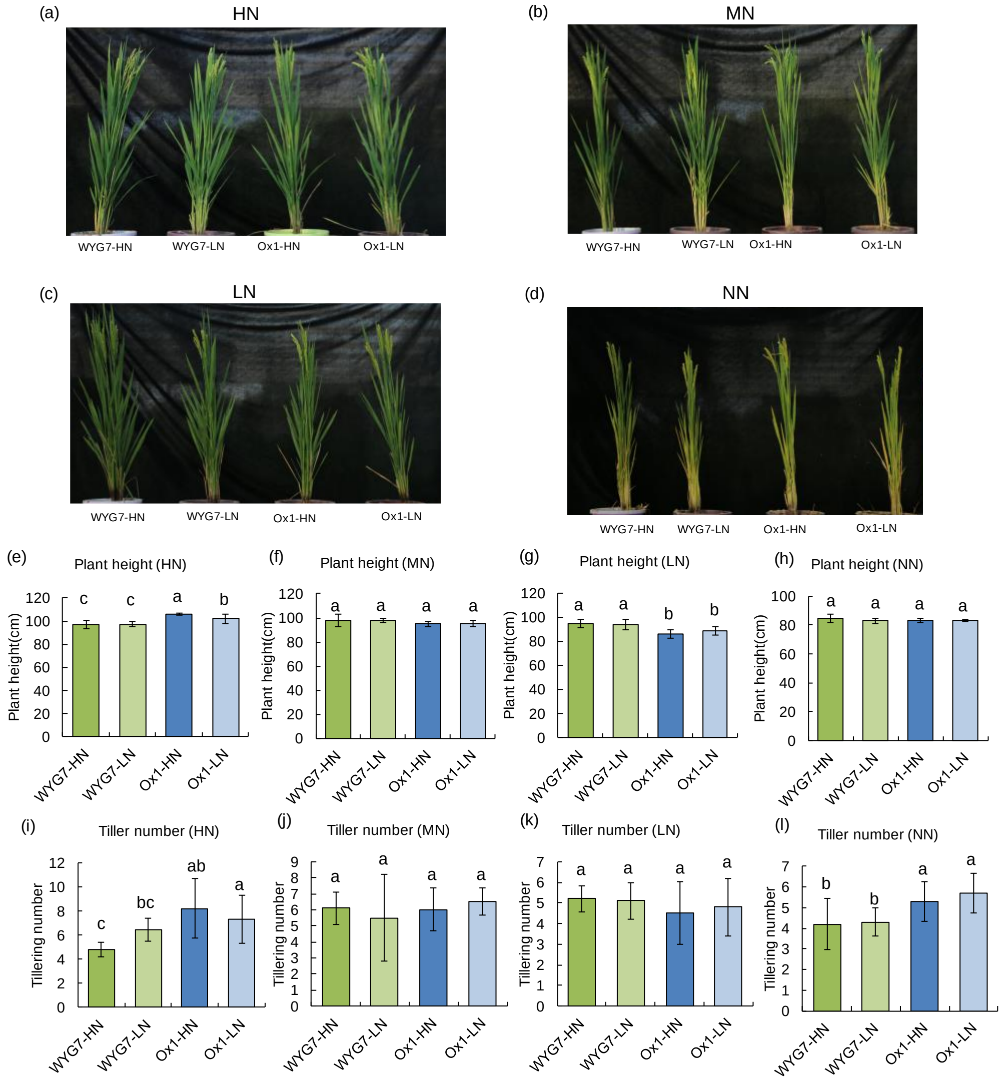


**Fig. S2. The decrease in the parent seed nitrogen content influence on phenotype**

(a-d) Gross morphology of different lines in HN, MN, LN and NN field. Bar = 10cm. (e-f) Plant height of different lines in HN, MN, LN and NN fertilizer field (i-l) Tiller number of different lines in HN, MN, LN and NN fertilizer field. Error bars: SD (n = 10). Significant differences between different lines are indicated by different letters ($P < 0.05$, one-way ANOVA).

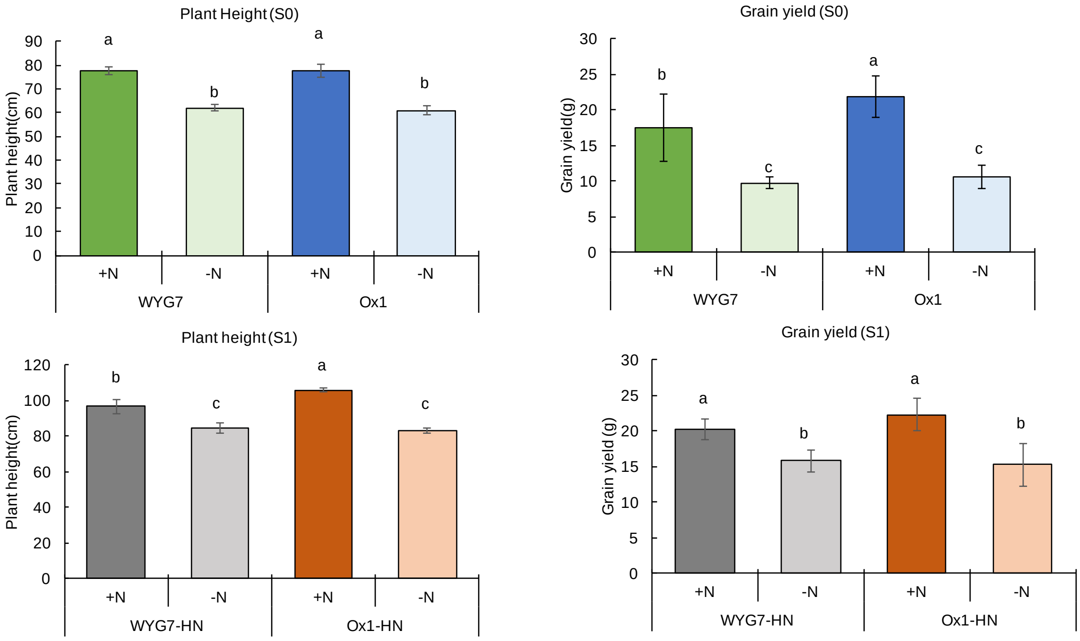


**Fig. S3. Plant height and grain yield induce by N deficiency in S0 and S1 generation.**

N deficiency influence on plant height of WYG7 and Ox1 in (a) S0 generation and (c) S1 generation. Error bar: SD (n=10 plants). N deficiency influence on grain yield per plant of WYG7 and Ox1 in (b) S0 generation and (d) S1 generation. Error bar: SD (n=5 plants).

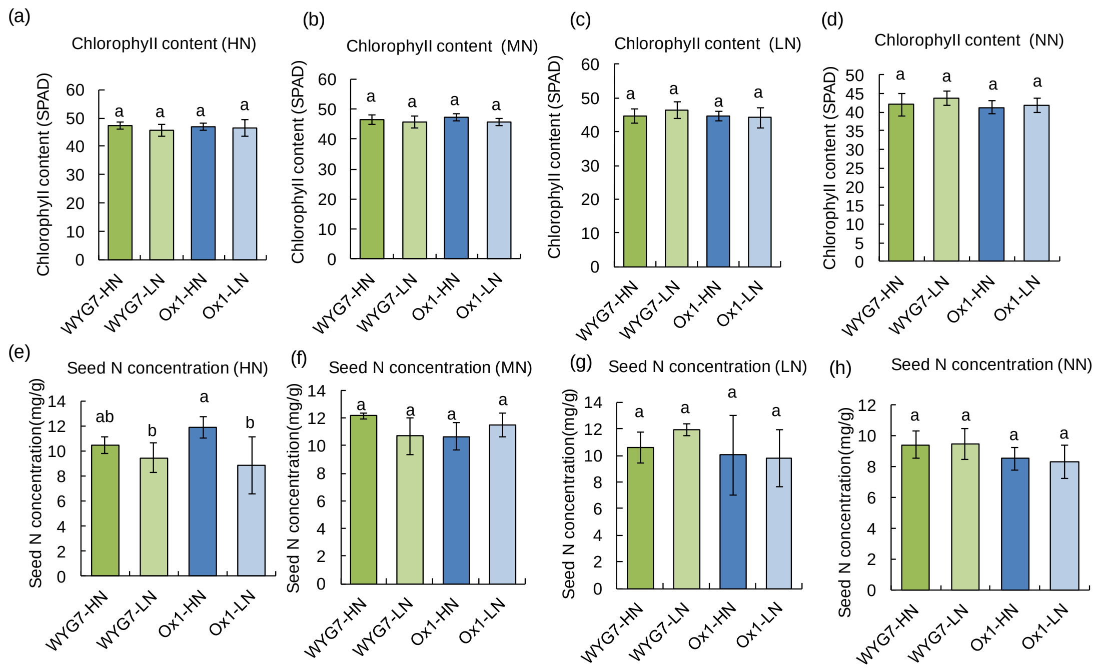


**Fig. S4. The decrease in the parent seed nitrogen content influence on chorophyII content and seed N concentration**

(a-d) ChorophyII content of WYG7-HN, WYG7-LN, Ox1-HN, Ox1-LN in HN, MN, LN and NN fertilizer field. Error bar: SD (n=5). (e-h) Filial seed N concentration of different lines in HN, MN, LN and NN fertilizer field. Error bars: SD (n = 5). Significant differences between different lines are indicated by different letters ($P < 0.05$, one-way ANOVA).

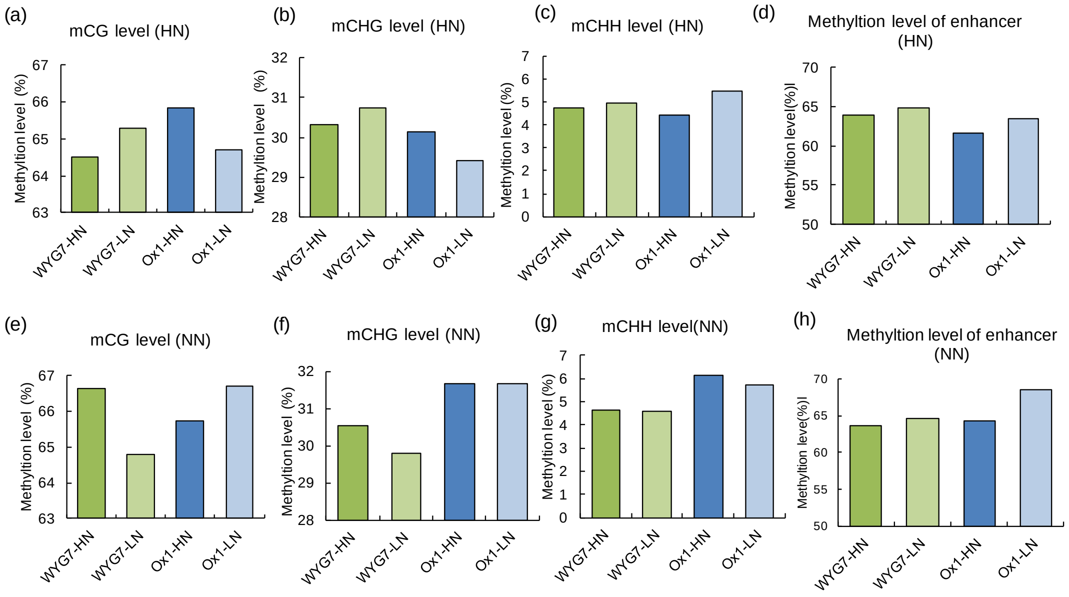


**Fig. S5. Methylation level of Ox1 and WYG7 lines**

(a-c) mCG level, mCHG level and mCHH level of different lines in HN field. (d) Methylation of enhancers in different lines in HN field. (e-g) mCG level, mCHG level and mCHH level of different lines in NN field. (h) Methylation of enhancers in different lines in NN field.

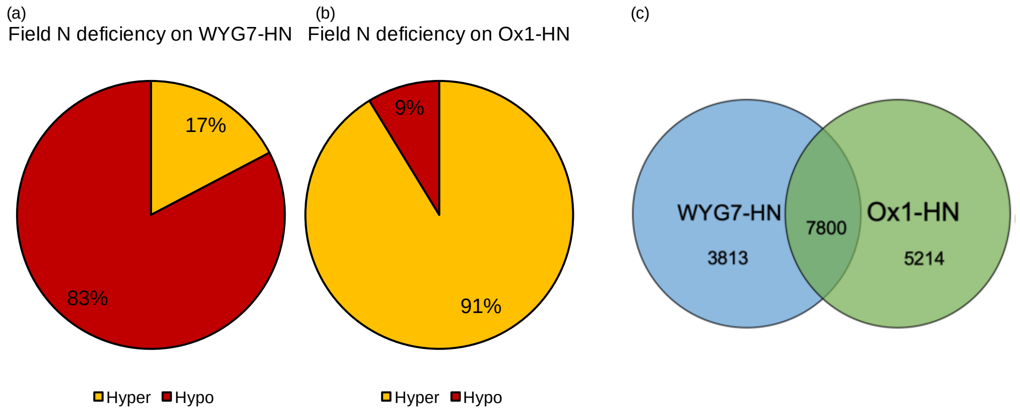


**Fig. S6. Differential methylation induces by field N deficiency in WYG7-HN and Ox1-HN**

(a, b) Breakdown of hyper- and hypo-DMRs in WYG7-HN and Ox1-HN induce by field N deficiency. (c) Venn diagram of unique and shared hyper- and hypo-DMGs in WYG7-HN and Ox1-HN induce by field N deficiency.

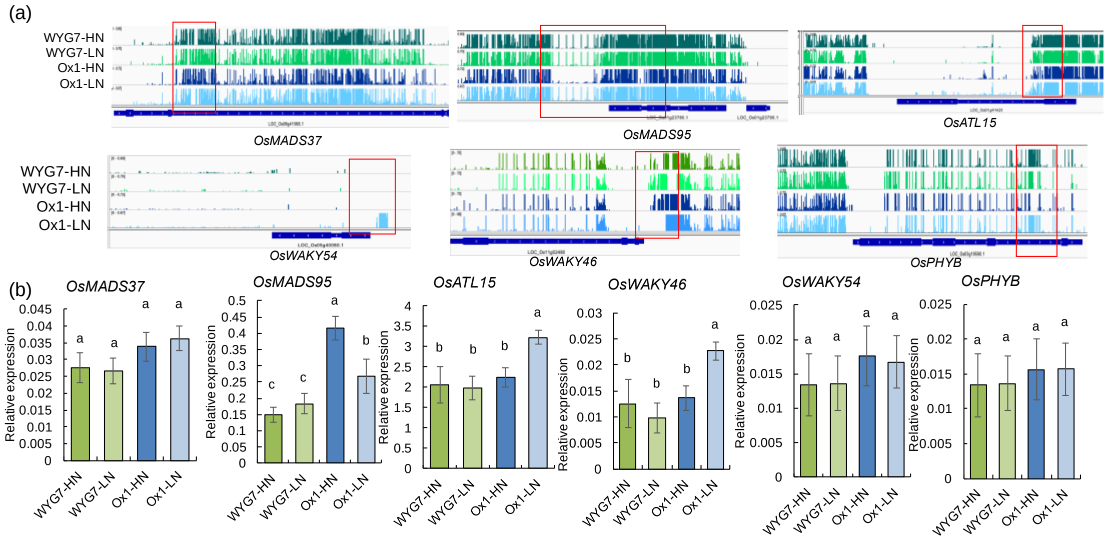


**Fig. S7. Methylation status and gene expression of relative genes in HN field**

(a) Methylation status of relative genes in HN field. (b) Gene expression of relative genes. Error bars: SD (n = 4 plants) in HN field. Significant differences between different lines are indicated by different letters ($P < 0.05$, one-way ANOVA)

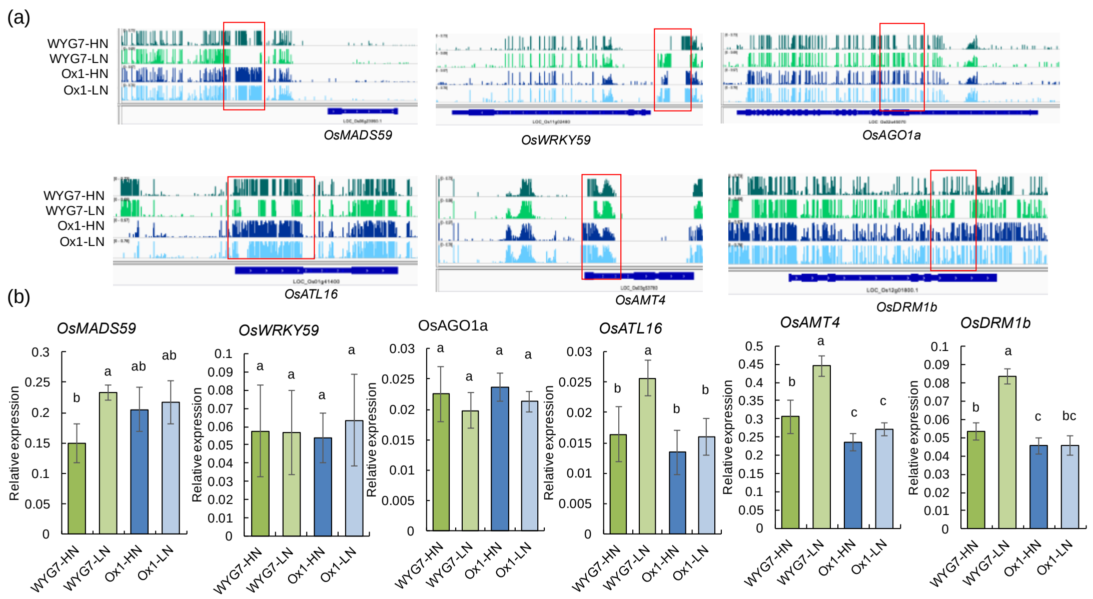


**Fig. S8. Methylation status and gene expression of relative genes in NN field**

(a) Methylation status of relative genes in NN field. (b) Gene expression of relative genes. Error bars: SD (n = 4 plants) in NN field. Significant differences between different lines are indicated by different letters ($P < 0.05$, one-way ANOVA)

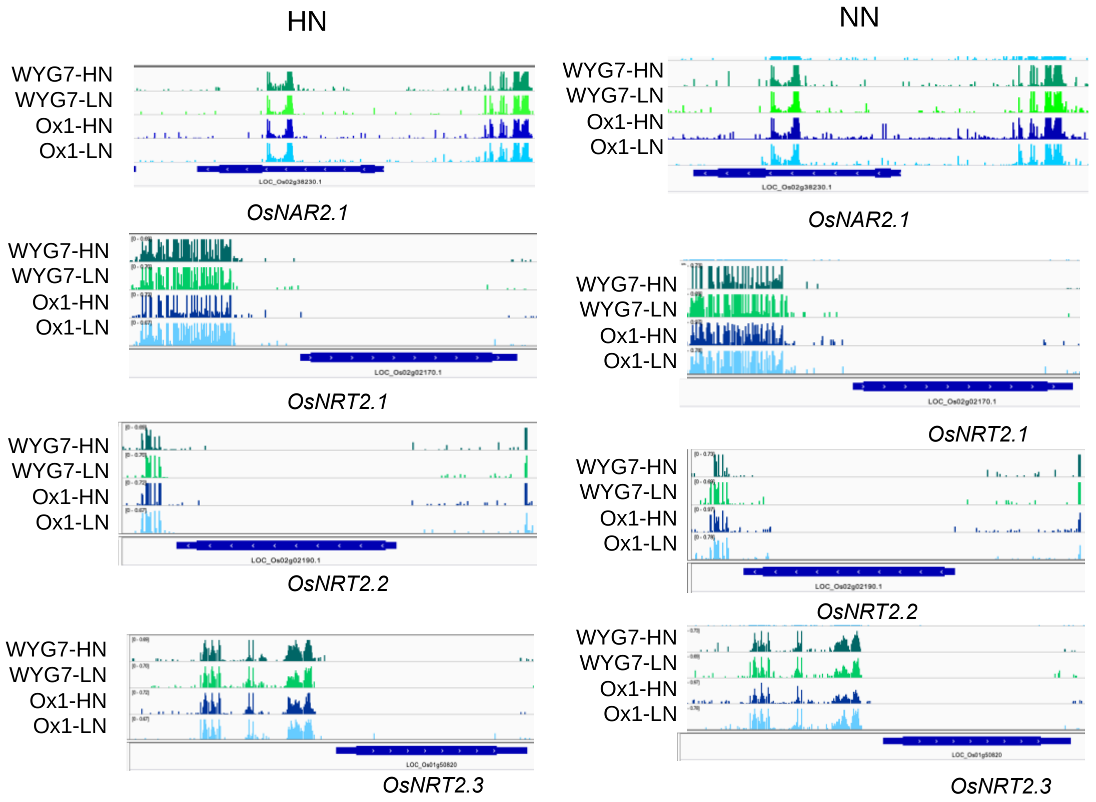


**Fig. S9. Methylation status of OsNRT family genes in different N field**

(a) Methylation status of OsNRT family genes in HN field. (b) Methylation status of OsNRT family genes in NN field.

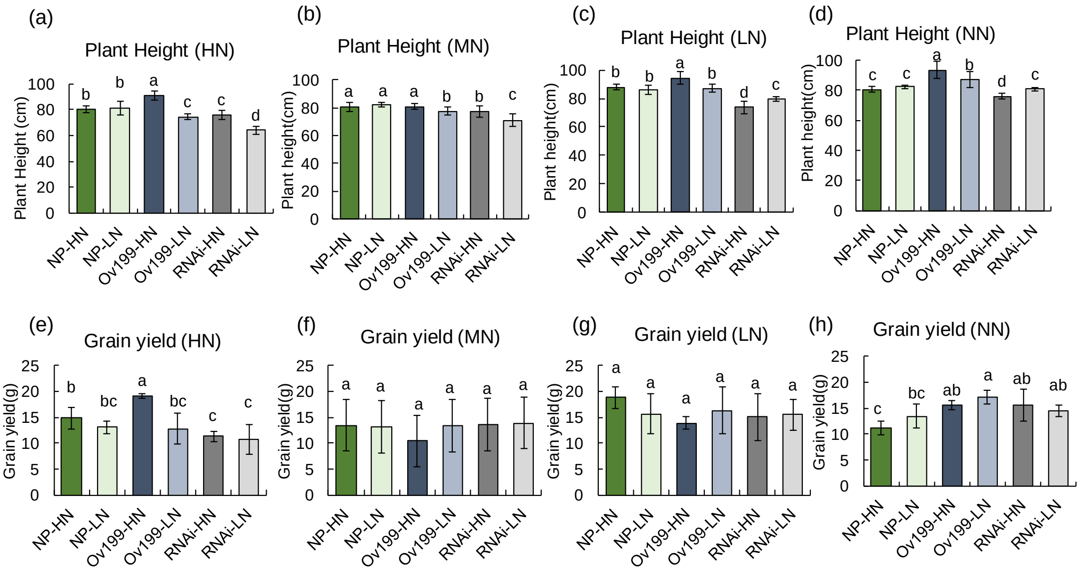


**Fig. S10. The decrease in parent seed nitrogen content of Ov199 leads to plant height and grain yield per plant decrease.**

(a-d) Plant height of different lines in the field with HN, MN, LN and NN fertilizer. Error bars: SD (n = 10). (e-h) Grain yield per plant of different lines in the field with HN, MN, LN and NN fertilizer. Error bars: SD (n = 5). Significant differences between different lines are indicated by different letters ($P < 0.05$, one-way ANOVA, Duncan).

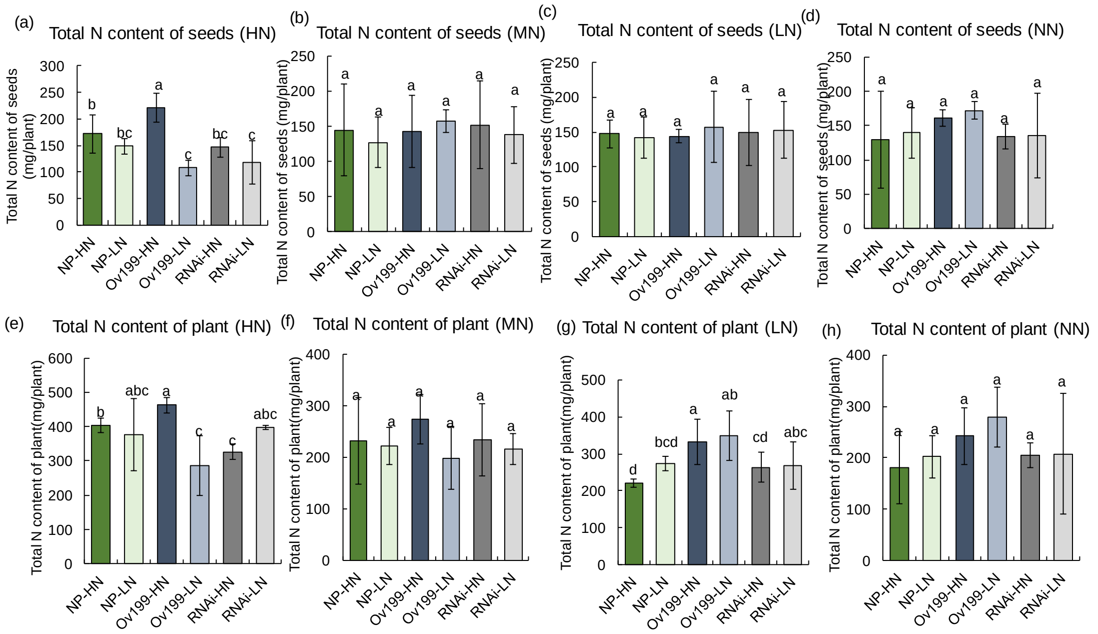


**Fig. S11. The decrease in parent seed nitrogen content of Ov199 leads to total N content of seeds and plant decrease**

(a-d) Total N content of filial seeds of different lines in the field with HN, MN, LN and NN fertilizer. Error bars: SD (n = 5). (e-h) Total N content of plant of different lines in the field with HN, MN, LN and NN fertilizer. Error bars: SD (n = 5). Significant differences between different lines are indicated by different letters ($P < 0.05$, one-way ANOVA, Duncan).

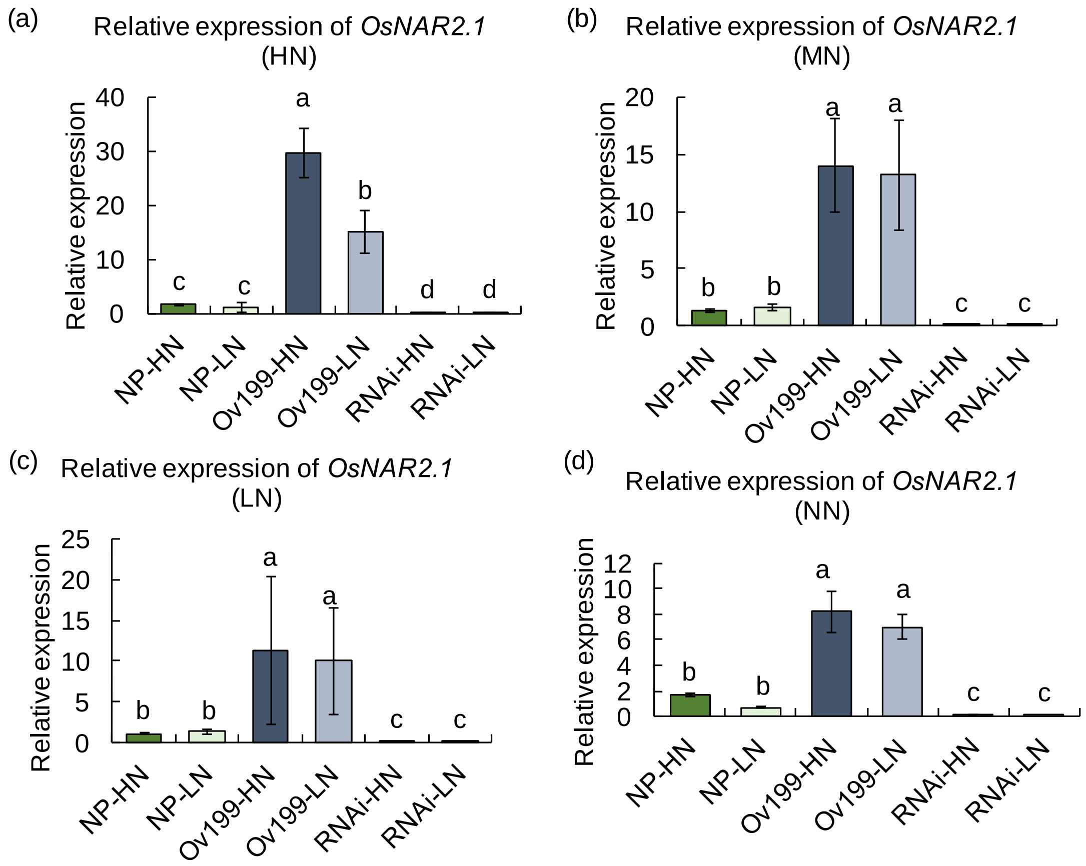


**Fig. S12. The decrease in parent seed nitrogen content of Ov199 leads to relative expression of *OsNAR2.1* decrease.**

The decrease in parent seed nitrogen content of Ov199 leads to relative expression of OsNAR2.1 decrease. (A-D) Relative expression of OsNAR2.1 of different lines in the field with HN, MN, LN and NN fertilizer. Error bars: SD (n = 4). Significant differences between different lines are indicated by different letters ($P < 0.05$, one-way ANOVA, Duncan).
